# Local, nonlinear effects of cGMP and Ca^2+^ reduce single photon response variability in retinal rods

**DOI:** 10.1371/journal.pone.0225948

**Published:** 2019-12-05

**Authors:** Giovanni Caruso, Vsevolod V. Gurevich, Colin Klaus, Heidi Hamm, Clint L. Makino, Emmanuele DiBenedetto

**Affiliations:** 1 CNR Ist Tecnologie Applicate ai Beni Culturali, Roma, Italy; 2 Department of Pharmacology, Vanderbilt University Medical Center, Nashville, Tennessee, Unites States of America; 3 The Mathematical Biosciences Institute, The Ohio State University, Columbus, Ohio, United States of America; 4 Department of Physiology and Biophysics, Boston University School of Medicine, Boston, Massachusetts, United States of America; 5 Department of Mathematics, Vanderbilt University, Nashville, Tennessee, United States of America; Doheny Eye Institute/UCLA, UNITED STATES

## Abstract

The single photon response (SPR) in vertebrate photoreceptors is inherently variable due to several stochastic events in the phototransduction cascade, the main one being the shutoff of photoactivated rhodopsin. Deactivation is driven by a random number of steps, each of random duration with final quenching occurring after a random delay. Nevertheless, variability of the SPR is relatively low, making the signal highly reliable. Several biophysical and mathematical mechanisms contributing to variability suppression have been examined by the authors. Here we investigate the contribution of local depletion of cGMP by PDE*, the non linear dependence of the photocurrent on cGMP, Ca^2+^ feedback by making use of a fully space resolved (FSR) mathematical model, applied to two species (mouse and salamander), by varying the cGMP diffusion rate severalfold and rod outer segment diameter by an order of magnitude, and by introducing new, more refined, and time dependent variability functionals. Globally well stirred (GWS) models, and to a lesser extent transversally well stirred models (TWS), underestimate the role of nonlinearities and local cGMP depletion in quenching the variability of the circulating current with respect to fully space resolved models (FSR). These distortions minimize the true extent to which SPR is stabilized by locality in cGMP depletion, nonlinear effects linking cGMP to current, and Ca^2+^ feedback arising from the physical separation of E* from the ion channels located on the outer shell, and the diffusion of these second messengers in the cytoplasm.

## Introduction

**Fig 1:**
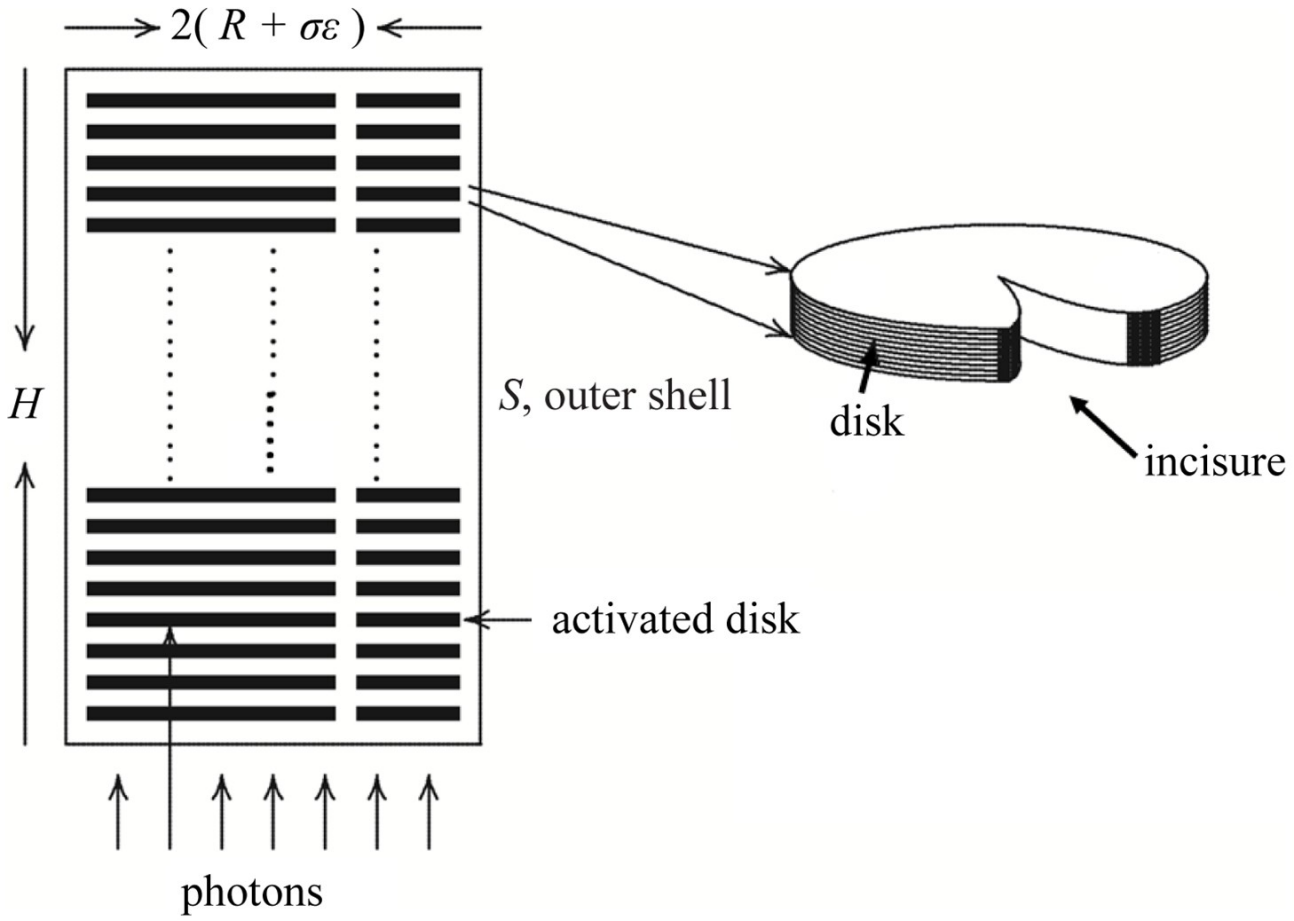
Structure of an idealized rod outer segment. Discs in mouse often have an incisure and within the outer segment, the incisures are in register, as shown in the figure. Salamander discs are larger with many more incisures. For modeling mouse rod, each disc had a single incisure, *H* was set to 23.6 *μ*m and (*R* + *σε*) was set to 0.7 *μ*m. For salamander, discs had 23 incisures, *H* was set to 22.4 *μ*m and (*R* + *σε*) was set to 5.515 *μ*m.

Vertebrate rod photoreceptors accurately detect light and reliably discriminate differences at exceedingly low levels of illumination. The biochemical cascade that transduces photons into integrated electrical signals that lead to changes in neurotransmitter release at the synapse is inherently stochastic. A photon, absorbed by the 11-cis-retinal covalently attached to rhodopsin, converts it into all-trans-retinal, which forces rhodopsin into an active state (R → R*). Active rhodopsin R* can be localized anywhere on the rod outer segment disc membrane (Fig 1), and it continues random diffusion in the membrane, encountering and activating a variable number of transducin G-protein (T) molecules by facilitating the exchange of GDP bound to inactive heterotrimeric transducin for GTP. Binding of GTP to the *α*-subunit of T induces its dissociation from active rhodopsin and from its *βγ*-subunit leaving R* free to activate additional molecules of transducin. The GTP-liganded T*α* (T*) physically interacts with a cGMP phosphodiesterase and activates it (E → E*). Active E* then hydrolyzes a variable number of cGMP molecules until it is deactivated as a result of the hydrolysis of GTP to GDP by the intrinsic GTPase activity of T*α* which is facilitated by both E ([1]) and RGS9 protein ([2]). Transducin deactivation is also a stochastic process, which makes E* lifetime stochastic. The resultant drop in cytoplasmic cGMP concentration leads to the closure of hundreds of cGMP-gated (CNG) ion channels on the plasma membrane, preventing the entry of Na^+^ ions into the rod. Channel closure also prevents the influx of Ca^2+^, whereas its efflux by a Ca^2+^-exchanger continues. This leads to a drop in cytoplasmic Ca^2+^ concentration ([3]). Calcium dissociates from guanylyl cyclase activating proteins (GCAPs) to be replaced by Mg^2+^, which converts GCAPs from inhibitors to activators of guanylyl cyclase (GC) ([4]). Increased GC activity then replenishes cytoplasmic cGMP. Synthesis of cGMP by GC is governed by a feedback system based on Ca^2+^. Activity is slow when Ca^2+^ is high in the dark, but accelerates when Ca^2+^ levels fall during the light response. As a result the channels reopen sooner, with a consequent rise of Ca^2+^, and eventual GC inactivation by Ca^2+^-liganded GCAPs. During response recovery R* is rapidly phosphorylated by rhodopsin kinase ([5]). Active rhodopsin R* can acquire as many as six (in mice and humans) or seven (in cows) attached phosphates before it encounters arrestin-1 by diffusion and is completely inactivated by arrestin-1 binding. Progressive rhodopsin phosphorylation reduces the efficiency of transducin activation ([6]). Thus R* exists for variable times in each of the phosphorylation states with different activities and is stochastically shut off by arrestin-1, typically after acquiring three or more attached phosphates.

The stochastic nature of every step in this biochemical cascade generates significant variability. Yet the rod’s single photon response (SPR) is significantly less variable that one would expect ([7]). Different mechanisms were proposed to underlie this unexpected reproducibility of SPR ([7–13]). In ([13]), a fully space resolved (FSR) biophysical model of phototransduction in rods, populated by experimentally tested parameters ([13, 14]), was used to identify the key factors that generate variability of the SPR and those that suppress it. Variability arises primarily from the random steps governing deactivation of the cascade, which generate differences in the spatial distributions and lifetimes for the activated Transducer-Effector complex, T*-E*. The ensuing diffusion of second messengers is deterministic. Variability of the SPR is reduced by (i) the localized depletion of cGMP within the rod outer segment (ROS) by T*-E*, (ii) the subsequent diffusion of cGMP and Ca^2+^ in the cytosol of the ROS, and (iii) the nonlinear relations linking ionic current to [cGMP] and [Ca^2+^]. The first two factors are intricately tied to the complex shape of the outer segment, which is conserved across species (Fig 1). The ROS houses a stack of disc structures, whose upper and lower membrane surfaces contain receptor rhodopsin R, G-protein transducer T and PDE effector E. While these components are mobile on the disc surface, they do not “hop” from disc to disc. Hence their spatial positions vary over time in transverse directions, but are fixed in the longitudinal direction. In contrast, the soluble cGMP substrate of E* moves throughout the cytoplasm of the ROS communicating E* activity on the surface of the activated disc, to CNG channels on the plasma membrane. The discs pose a barrier to movement of cGMP, but they typically contain one or more incisures that facilitate longitudinal diffusion of soluble second messengers.

Because of the small size of the transversal cross section of the ROS, with respect to its length in most species, it is tempting to assume that transversal cGMP diffusion plays a negligible role in the cascade, all the more in the presence of a large diffusion coefficient D_cG_, which would favor rapid transversal equilibration ([15, 16]). The relatively small changes in [cGMP] that occur during the SPR invite a further simplification, linearization of the relation between [cGMP] and ion channel activity ([15, 16]). We disprove such approaches by providing numerical evidence that the full 3-dimensional structure of the ROS and the *local* nature of the cascade activation-deactivation and nonlinearities in the effects of second messengers, play key roles in suppressing variability. To underscore the role of localization and to extract the contribution of diffusion, the simulations were effected for small diameter mouse rods and for large diameter salamander rods, and for several values of D_cG_. The role of Ca^2+^ feedback was evaluated by “clamping” [Ca^2+^] at the dark level. The suppressive effects of locality, nonlinearity and Ca^2+^ feedback on SPR variability change in significance over time, an aspect that is largely ignored by the functionals usually used to assess SPR variability. Here we discuss what information is provided by different functionals that evolve over time and use them to show how variability is distorted by reducing spatial resolution and by linearization of second messenger effects.

## Materials and methods

The current densities J_ex_ and J_cG_ due to Ca^2+^ exchange and the cGMP-gated channels, respectively, are given by

$$\begin{array}{c} {\text{J}}_{\text{ex}}=\frac{{\text{j}}_{\text{ex}}^{\text{sat}}}{\Sigma_{\text{rod}}}\frac{[\text{Ca}{}^{2+}\text{]}}{{\text{K}}_{\text{ex}}+[\text{Ca}{}^{2+}\text{]}}\;\;\text{on}\;\;S, \end{array}$$ (1)

$$\begin{array}{c} {\text{J}}_{\text{cG}}=\frac{{\text{j}}_{\text{cG}}^{\text{max}}}{\Sigma_{\text{rod}}}\;\frac{{\left[\text{cGMP}\right]}^{{\text{m}}_{\text{cG}}}}{{\text{K}}_{\text{cG}}^{{\text{m}}_{\text{cG}}}+{\left[\text{cGMP}\right]}^{{\text{m}}_{\text{cG}}}}\;\;\text{on}\;\;S. \end{array}$$ (2)

In Eq 1, ${\text{j}}_{\text{ex}}^{\text{sat}}$ is the saturated exchange current (as [Ca^2+^] → ∞), K_ex_ is the Ca^2+^ concentration at which the exchange rate is half maximal, and Σ_rod_ is the surface area of the lateral boundary of the ROS. In Eq 2, ${\text{j}}_{\text{cG}}^{\text{max}}$ is the maximal cGMP-current (as [cGMP] → ∞), m_cG_ is the Hill exponent, and K_cG_ is the half-maximal channel opening concentration of cGMP. These formulae are “local” as they provide the current densities in terms of space-time values of [cGMP] and [Ca^2+^] computed on the lateral boundary *S*, of the ROS. In the absence of light, J_ex_ and J_cG_ are constant and equal to their “dark” values J_ex;dark_ and J_cG;dark_ defined as in Eqs 1 and 2 with [Ca^2+^] and [cGMP] replaced by [Ca^2+^]_dark_ and [cGMP]_dark_, respectively. The current J_tot_(*x*, *y*, *t*) at (*x*, *y*) ∈ *S* at time *t*, and the total current j_tot_, across the whole lateral boundary of the ROS, are

$$\begin{array}{c} J_{\text{tot}}={\text{J}}_{\text{ex}}+{\text{J}}_{\text{cG}};\;{\text{j}}_{\text{tot}}\left(t\right)=\int_{S}J_{\text{tot}}\left(x,y,t\right)dS \end{array}$$ (3)

where *dS* is the surface measure on *S*. The experimentally measured electrophysiological response, is the current suppression relative to its dark value, i.e.,

$$\begin{array}{c} \text{I}\left(t\right)=1-\frac{{\text{j}}_{\text{tot}}\left(t\right)}{{\text{j}}_{\text{dark}}}. \end{array}$$ (4)

### Measuring the variability of the single photon response

Variability of the SPR may be measured by the coefficient of variation (CV), defined as the standard deviation over the mean of a pre-chosen functional:

$$\begin{array}{c} {\text{I}}_{\text{int}}\left(t\right)=\int_{0}^{t}I\left(s\right)ds,\;{\text{I}}_{\text{area}}=\int_{0}^{\infty }I\left(t\right)dt,\;\text{I}\left({\text{t}}_{\text{peak}}\right),\;{\text{j}}_{\text{tot}}\left(t\right). \end{array}$$ (5)

The first is the total relative charge suppression up to time *t*, the second is the total charge suppression over the entire time course of the SPR, the third is the current suppression I(*t*) when the response is maximal, i.e., at time t_peak_, and the last one *t* → j_tot_(*t*), as defined by Eq 3, is the dynamic of the total *actual current* across the outer shell of the ROS along the time course of the process. The CVs for these functionals, with the exception of the last one, have appeared in the literature as a measure of SPR variability ([7–13, 13, 15–20]). The integral quantities I_int_(*t*) and I_area_ are regarded as suitable because “*the area captures fluctuations occurring at any time during the response, and thus provides a good measure of the total extent of response fluctuations*” ([9]). Pointwise fluctuations are tracked by I(*t*) and hence I(t_peak_) ([11, 15, 16]). Which of these best measures the randomness of the SPR, remains elusive.

The relevance of the information contained in the CV of these functionals may change according to the way the signal is processed downstream of the cascade. At the limit of sensitivity, the bipolar cell synapse ignores low amplitude phototransduction noise but may be saturated by the rod SPR. In this case, variability in SPR amplitude may not have functional consequences. The bipolar cell response is also faster than the rod response; the bipolar cell response is largely complete by the time the rod response reaches its peak, so it would seem that rod response recovery is not significant either. Since the functional I_area_ is dominated by the recovery phase of the rod SPR, CV(I_area_) might not be that important. On the other hand, Field et al ([21]) suggest that saturation at the bipolar cell may occur with more than one photon, in which case rod SPR amplitude I(t_peak_) and integration time I_int_(*t*) as well as their variability become important. It is possible that both scenarios are correct under different adaptation regimes; Taylor et al ([22]) may be describing the situation under the most dark adapted conditions, whereas Field et al ([21]) may be looking at it under very slightly light adapted conditions. To complicate matters further, rods are electrically coupled to other rods and cones, enabling the SPR from one rod to spread across the retina. But like ripples in a pond disturbed by a stone dropping into the water, the signal diminishes with distance from the rod generating the SPR. So under slightly light adapted conditions, SPR amplitude and area and their variability again become important. The CV of the functional j_tot_(*t*), to the best of our knowledge has not been used in the literature. Yet it seems to be a relevant functional for the reasons we present below.

Let *X* be the probability space of events of I(*t*) with probability measure *dω*. Then from Eqs 3 and 4

$$\begin{array}{c} \begin{array}{cc} \text{CV}[\text{I}(t)] & =\frac{\sqrt{\int_{X}(\text{I}\left(t;\omega \right)-\int_{X}\text{I}\left(t;\omega \right)d\omega {)}^{2}d\omega }}{\int_{X}\text{I}\left(t;\omega \right)d\omega }\\ & =\frac{\sqrt{\int_{X}[(1-\frac{{\text{j}}_{\text{tot}}\left(t;\omega \right)}{{\text{j}}_{\text{dark}}})-\int_{X}(1-\frac{{\text{j}}_{\text{tot}}\left(t;\omega \right)}{{\text{j}}_{\text{dark}}})d\omega {]}^{2}d\omega }}{\int_{X}(1-\frac{{\text{j}}_{\text{tot}}\left(t;\omega \right)}{{\text{j}}_{\text{dark}}})d\omega }\\ & =\frac{\sqrt{\int_{X}({\text{j}}_{\text{tot}}\left(t;\omega \right)-\int_{X}{\text{j}}_{\text{tot}}\left(t;\omega \right)d\omega {)}^{2}d\omega }}{\int_{X}({\text{j}}_{\text{dark}}-{\text{j}}_{\text{tot}}\left(t;\omega \right))d\omega }. \end{array} \end{array}$$ (6)

While starting with the current drop I(*t*), the numerator of this fraction is the standard deviation of j_tot_(*t*) and not its relative drop. Normalizing the numerator by the probabilistic mean of j_tot_(*t*), gives the CV of j_tot_(*t*), i.e.,

$$\begin{array}{c} \text{CV}[{\text{j}}_{\text{tot}}\left(t\right)]=\frac{\sqrt{\int_{X}({\text{j}}_{\text{tot}}\left(t;\omega \right)-\int_{X}{\text{j}}_{\text{tot}}\left(t;\omega \right)d\omega {)}^{2}d\omega }}{\int_{X}{\text{j}}_{\text{tot}}\left(t;\omega \right)d\omega }. \end{array}$$ (7)

Combining these formulae yields a relation between CV[I(*t*)] and CV[j_tot_(*t*)], of the form

$$\begin{array}{c} \text{CV}\left[\text{I}\left(t\right)\right]=\text{CV}\left[{\text{j}}_{\text{tot}}\left(t\right)\right]\;\frac{\int_{X}{\text{j}}_{\text{tot}}\left(t;\omega \right)d\omega }{\int_{X}({\text{j}}_{\text{dark}}-{\text{j}}_{\text{tot}}\left(t;\omega \right))d\omega }. \end{array}$$ (8)

As *t* → ∞, the system returns to its dark adapted steady state. Since the models make no provisions for “dark noise” in the phototransduction system there are no further fluctuations, and variability reduces to zero. Indeed, for CV[j_tot_(*t*)] as defined by Eq 7, the denominator remains “close” to its dark value whereas the numerator goes to zero, so that the corresponding CV then goes to zero as *t* → ∞. But for CV[I(*t*)] as defined by Eq 6, both numerator and denominator go to zero, yielding a non-zero asymptotic value for the corresponding CV (for example, see §,§).

A further justification for considering CV of J_tot_(*t*) is that transmission of the SPR at the synapse, downstream of the cascade, is concerned with voltage change, which depends upon j_tot_(*t*) and not the relative current drop I(*t*). The latter is a contrived way to avoid referring to a decrease in an inward cationic current in response to photon absorption. We will use the dynamics of the functional *t* → j_tot_(*t*) and its CV in the context of Ca^2+^ clamped virtual experiments.

### Diffusion of cGMP and Ca^2+^ in the cytoplasm

Following ([11, 23, 24]), in the cytoplasm [cGMP] satisfies the diffusion equation

$$\begin{array}{c} {}[\text{cGMP}{]}_{t}-{\text{D}}_{\text{cG}}\Delta_{\bar{x}}[\text{cGMP}]=F-\delta_{z_{*}}\frac{{\text{k}}_{\sigma ;\text{hyd}}^{*}}{\nu \varepsilon_{o}}{\left[{\text{E}}^{*}\right]}_{\sigma }[\text{cGMP}]. \end{array}$$ (9)

Here D_cG_ is the diffusion coefficient of cGMP in the cytosol and $\Delta_{\bar{x}}$ denotes the Laplacian with respect to the transversal variable $\bar{x}=\left(x,y\right)$ only. These are diffusion processes, parametrized with *z* ∈ (0, *H*), taking place on the homogenized horizontal layers of the ROS cytoplasm. Activation occurs at the level *z* = z_*_ and $\delta_{z_{*}}$ is the Dirac mass in *z* concentrated at *z* = z_*_. The coefficient ${\text{k}}_{\sigma ;\text{hyd}}^{*}$ is the surface hydrolysis rate (in *μ*m^3^s^−1^/#) of cGMP by the surface density of [E*]_*σ*_. The parameter *ε*_*o*_ is the thickness of the discs and *νε*_*o*_ is the width of the interdiscal spaces. The term *F* is given by

$$\begin{array}{c} F=(\alpha_{\text{min}}+\frac{\alpha_{\text{max}}-\alpha_{\text{min}}}{1+{\left([\text{Ca}{}^{2+}\text{]}/{\text{K}}_{\text{cyc}}\right)}^{{\text{m}}_{\text{cyc}}}})-\beta_{\text{dark}}[\text{cGMP}]. \end{array}$$ (10)

The first term in round brackets in the definition of *F* represents the production of cGMP by GC, which is located on the faces of the discs. Here *α*_max_ and *α*_min_ are positive constants with dimensions *μ*Ms^−1^. The last term in Eq 10 represents cGMP depletion due to hydrolysis of cGMP by PDE at a basal rate *β*_dark_. This process occurs at the faces of the discs and it involves the surface concentration of E through a surface hydrolysis rate k_*σ*;hyd_. The dynamic of Ca^2+^ is described by the equation

$$\begin{array}{c} {}[\text{Ca}{}^{2+}{\text{]}}_{t}-{\text{D}}_{\text{Ca}}\Delta [\text{Ca}{}^{2+}\text{]}=0\;\;\text{in}\;\text{the}\;\text{cytoplasm} \end{array}$$ (11)

complemented by the flux condition of the lateral boundary *S* of the ROS

$$\begin{array}{c} -{\text{D}}_{\text{Ca}}\nabla [\text{Ca}{}^{2+}\text{]}\cdot n=\eta ({\text{J}}_{\text{ex}}-\frac{1}{2}{\text{f}}_{\text{Ca}}{\text{J}}_{\text{cG}}). \end{array}$$ (12)

Here D_Ca_ is the diffusivity of Ca^2+^ in the cytoplasm, **n** is the outward unit normal to *S*, the currents J_ex_ and J_cG_ are defined in Eqs 1 and 2, *η* (in *n*m) is a positive parameter and f_Ca_ is the fraction of current carried by Ca^2+^ through the CNG channels (see B of S1 Appendix, for the meaning and values of these parameters).

Both Eqs 9, 10, 11 and 12 might not have a pointwise mathematical meaning and must be interpreted in a weak-integral form. They also need to be complemented by similar processes on the incisures and coupled equations on the lateral boundary of the ROS. A full, rigorous formulation of the model is in A.2—A.3 of S1 Appendix.

### The activation-deactivation cascade

Molecules of T* and E* diffuse on the activated disc by the random walk *t* → **x**(*t*) (dimension *μ*m^−2^) of R* on the activated disc *D* from which the limiting incisure has been removed. Following activation, R* becomes deactivated after an exponentially distributed random time t_R*_, of mean *τ*_R*_. During the random interval (0, t_R*_), R* evolves through *n* molecular states, produced by sequential R* phosphorylation by rhodopsin kinase, $\text{R}{}_{j}^{*}$, *j* = 1, …, *n*, each with transducer-activation rate *ν*_*j*_, with random transition times *t*_*j*−1_ < *t*_*j*_ ≤ t_R*_. Thus the rate equations for the surface densities [E*]_*σ*_ and [T*]_*σ*_ are

$$\begin{array}{c} \begin{array}{cc} & {{\left[{\text{T}}^{*}\right]}_{\sigma }}_{t}-{\text{D}}_{\text{T}}\Delta {\left[{\text{T}}^{*}\right]}_{\sigma }=\overset{n}{\underset{j=1}{\sum }}\nu_{j}\chi_{\left(t_{j-1},t_{j}\right]}\left(t\right)\delta_{x(t)}-{\text{k}}_{{\text{T}}^{*}\text{E}}{\left[\text{E}\right]}_{\sigma }{\left[{\text{T}}^{*}\right]}_{\sigma }\\ & {{\left[{\text{E}}^{*}\right]}_{\sigma }}_{t}-{\text{D}}_{\text{E}}\Delta {\left[{\text{E}}^{*}\right]}_{\sigma }={\text{k}}_{{\text{T}}^{*}\text{E}}{\left[\text{E}\right]}_{\sigma }{\left[{\text{T}}^{*}\right]}_{\sigma }-{\text{k}}_{\text{E}}{\left[{\text{E}}^{*}\right]}_{\sigma } \end{array} \end{array}$$ (13)

in *D*, complemented by homogeneous initial data, and no-flux boundary conditions on the boundary of *D*. Here k_T*E_ is the coupling coefficient from T* to E*. The constant k_E_ is the rate of deactivation of T* within the T*-E* complex. The constants *ν*_*j*_ are the rate of activation of T* by $\text{R}{}_{j}^{*}$ through a successful encounter at time *t* ∈ (*t*_*j*−1_, *t*_*j*_]. The times *s*_*j*_ = (*t*_*j*_ − *t*_*j*−1_) are exponentially distributed random sojourn times of R* in its j*th* phosphorylation state. The method is introduced in detail in ([12]), along with the corresponding parameters, which are reported in B.1.1 of S1 Appendix, and in B.2.1 of S1 Appendix for salamander.

These equations also need to be mathematically interpreted in a weak form (A.4 of S1 Appendix). The FSR model, its mathematical weak formulation, and its biological validation have been introduced and refined in a series of contributions ([11–14, 23–27]). The main equations are reproduced here in a pointwise form, to stress that the only source of variability in the cascade is the surface dynamics of T* and E* on the activated disc, as expressed by the system of Eq 13. The function [cGMP] experiences randomness only through the random term [E*]_*σ*_, which appears on the right-hand side of Eq 9. Then [cGMP] is computed and put to use sequentially in Eqs 2–5.

Let E*(*t*) denote the total number of molecules of activated effector PDE* at time *t*, downstream of the activation-deactivation cascade, i.e.,

$$\begin{array}{c} {\text{E}}^{*}\left(t\right)=\int_{D}{\left[{\text{E}}^{*}\right]}_{\sigma }\left(x,y,t\right)dxdy. \end{array}$$ (14)

The variability of [E*]_*σ*_ is computed by the variability of the functionals

$$\begin{array}{c} {\text{E}}_{\text{int}}^{*}\left(t\right)=\int_{0}^{t}{\text{E}}^{*}\left(s\right)ds,\;{\text{E}}_{\text{area}}^{*}=\int_{0}^{\infty }{\text{E}}^{*}\left(t\right)dt,\;{\text{E}}_{\text{max}}^{*}. \end{array}$$ (15)

The first is the activity of E* up to time *t*, the second is the total activity of E* produced over the entire lifetime of the process and the last is the maximum of E*(*t*) at its peak time ${\text{t}}_{\text{peak}}^{*}$. While these functionals directly express the randomness of the activation/deactivation cascade, to our knowledge, they are not experimentally accessible in intact rods.

These functionals parallel at the activation/deactivation level, the functionals I_int_(*t*), I_area_, I(t_peak_), and j_tot_(*t*) at the response level, as defined in Eqs 3–5. Notice that ${\text{t}}_{\text{peak}}\neq {\text{t}}_{\text{peak}}^{*}$. Randomness of the experimentally measured current suppression I_int_(*t*) and I_area_ is indirectly imported from E* through [cGMP], by the Eqs 9 and 2. To separate these two levels of randomness in our simulations we report the time dynamic of the $\text{CV}({\text{E}}_{\text{int}}^{*}\left(t\right))$ alongside with the CV(I_int_(*t*)), to highlight how the latter reflects a variability reduction of the former. Numerical experiments are performed on mouse and salamander rods, for which there are complete and consistent sets of parameters ([11–14]) (see B of S1 Appendix). The cGMP diffusion coefficient for mouse was taken as D_cG_ = 120 *μ*m^2^/s, close to the reported experimental value D_cG_ ≈ 140 *μ*m^2^/s ([28]). In our mouse simulations we also tested a theoretical value, D_cG_ = 330 *μ*m^2^/s suggested in ([29]) and used in simulations for the transversally well-stirred model of ([15, 16, 30]). By the conversion formula of ([24]) (D of S1 Appendix) these correspond to *longitudinal* diffusion coefficients of ≈ 14 *μ*m^2^/s and ≈ 40*μ*m^2^/s. Using D_cG_ = 330 *μ*m^2^/s and keeping the remaining parameters unchanged reproduces the experimental SPR presented in ([12]), for the choice *ν*_RG_ ≈ 230/s (C of S1 Appendix). This new value yields new catalytic activities *ν*_*j*_ for R* in its *j*th phosphorylated state by the formula *ν*_*j*_ = *ν*_RG_ exp{−*k*_*v*_(*j* − 1)} ([12]). For the salamander simulations we include 23 radially equally spaced incisures assimilated to right circular sectors with “base” 15nm set on the rim of the discs, radius 4.64 *μ*m, and angle 0.015/4.64 radians. The total area of the incisures is 0.8 *μ*m^2^.

### Virtual experiments

#### Fully space resolved versus transversally and globally well stirred models

Our FSR model takes into account all geometrical aspects of the ROS. The TWS model assimilates the ROS to a segment of length *H*, thereby disregarding the transversal dynamic of the players, and more importantly the intricate relationship between interior and boundary dynamics. While at times used, in view of its mathematical simplicity ([15, 16, 31]), no justification is provided other than it is “generally accepted” because of transversal “rapid equilibration”. The GWS model removes the geometry of ROS altogether and describes the cascade only as a sequence of mass action relations of the various players. The TWS and GWS models can be derived by Eqs 1–13, and their weak formulations (A.3 of S1 Appendix) by progressively removing the geometrical features of the ROS. The limitations of such lumped models have been presented in ([11, 13, 23, 24]). The GWS model is equivalent to letting D_cG_ → ∞ in the FSR model, so the effect of diffusion was explored by testing several values for D_cG_. The TWS model is approached by the FSR as disc diameter is reduced to zero, so to assess the effect of disc size separately, we simulated mouse and salamander rods, because their disc sizes span the range found in nature. In addition these are the two species for which the most knowledge has accumulated.

#### Local cGMP suppression and variability by deterministic simulations

Some initial simulations were carried out in which R* deactivation was deterministic to compare the response for short and long R* lifetimes. Photoexcitation of R was always taken as the center of the disc located in the middle of the ROS. The relative, local cGMP depletion and its average on the outer shell are

$$G\left(\rho ,\theta ,z,t\right)\;\underset{=}{\text{def}}\;1-\frac{\left[\text{cGMP}\right]\left(\rho ,\theta ,z,t\right)}{{\left[\text{cGMP}\right]}_{\text{dark}}};\;G_{S}\left(t\right)=\frac{1}{\left|\Sigma_{\text{rod}}\right|}\int_{S}G\left(t\right)dS.$$ (16)

**Fig 2:**
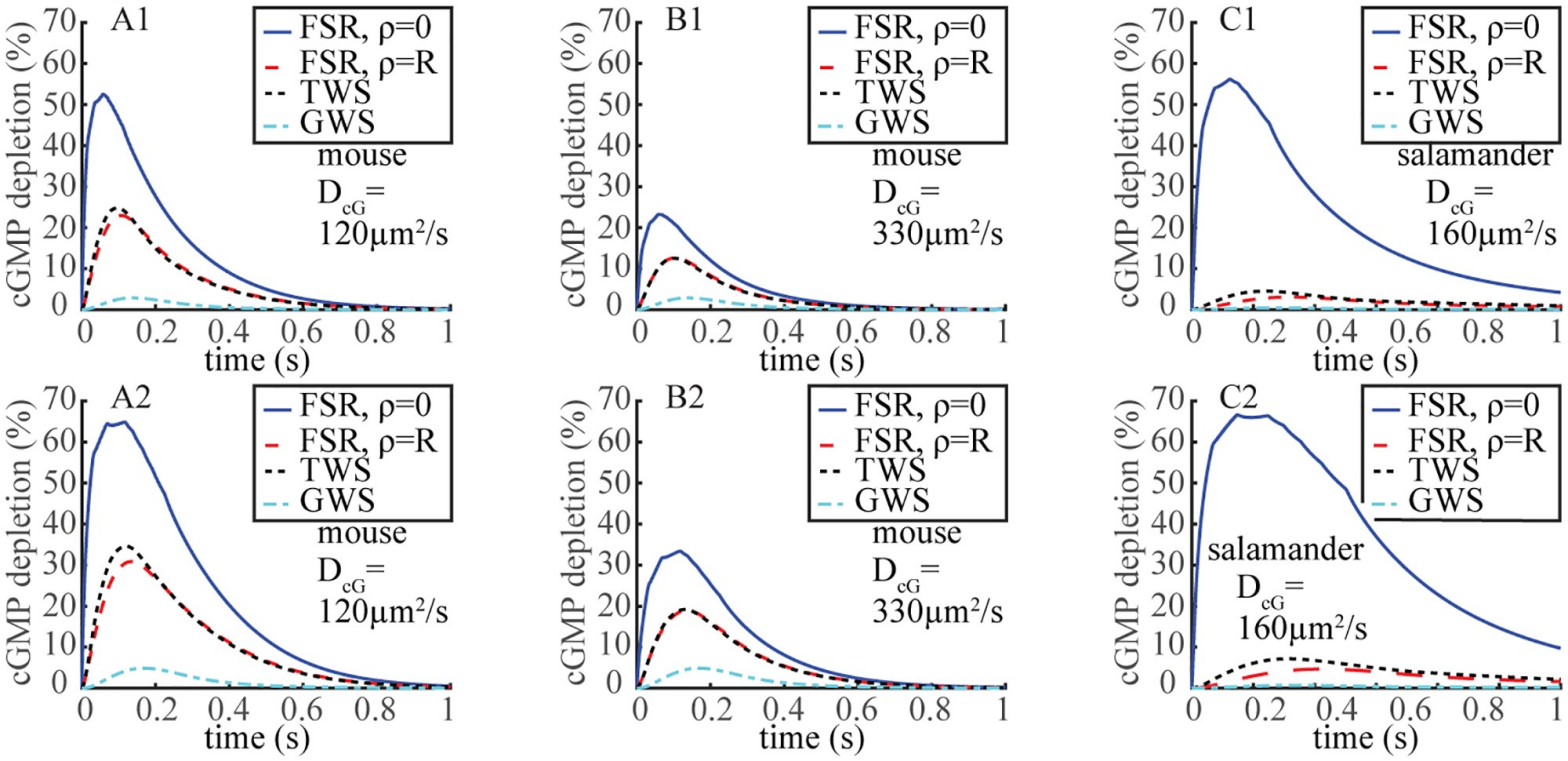
Spatial inhomogeneity of cGMP depletion across the activated disc during the SPR revealed by the FSR model. Time dynamics t→G(ρ,θ,z,t) of relative [cGMP](*ρ*, *θ*, *z*, *t*) depletion, as defined in Eq 16, at the activation site *ρ* = 0 and on the rim *ρ* = *R*, for $z=\frac{1}{2}H$. For these simulations deactivation of R* was deterministic with lifetime *τ*_av_ = 0.11 s (**A1, B1**) or 2*τ*_av_ = 0.22 s (**A2, B2**) for mouse and *τ*_av_ = 0.625 s (**C1**) or 2*τ*_av_ = 1.25 s (**C2**) for salamander.

**Fig 3:**
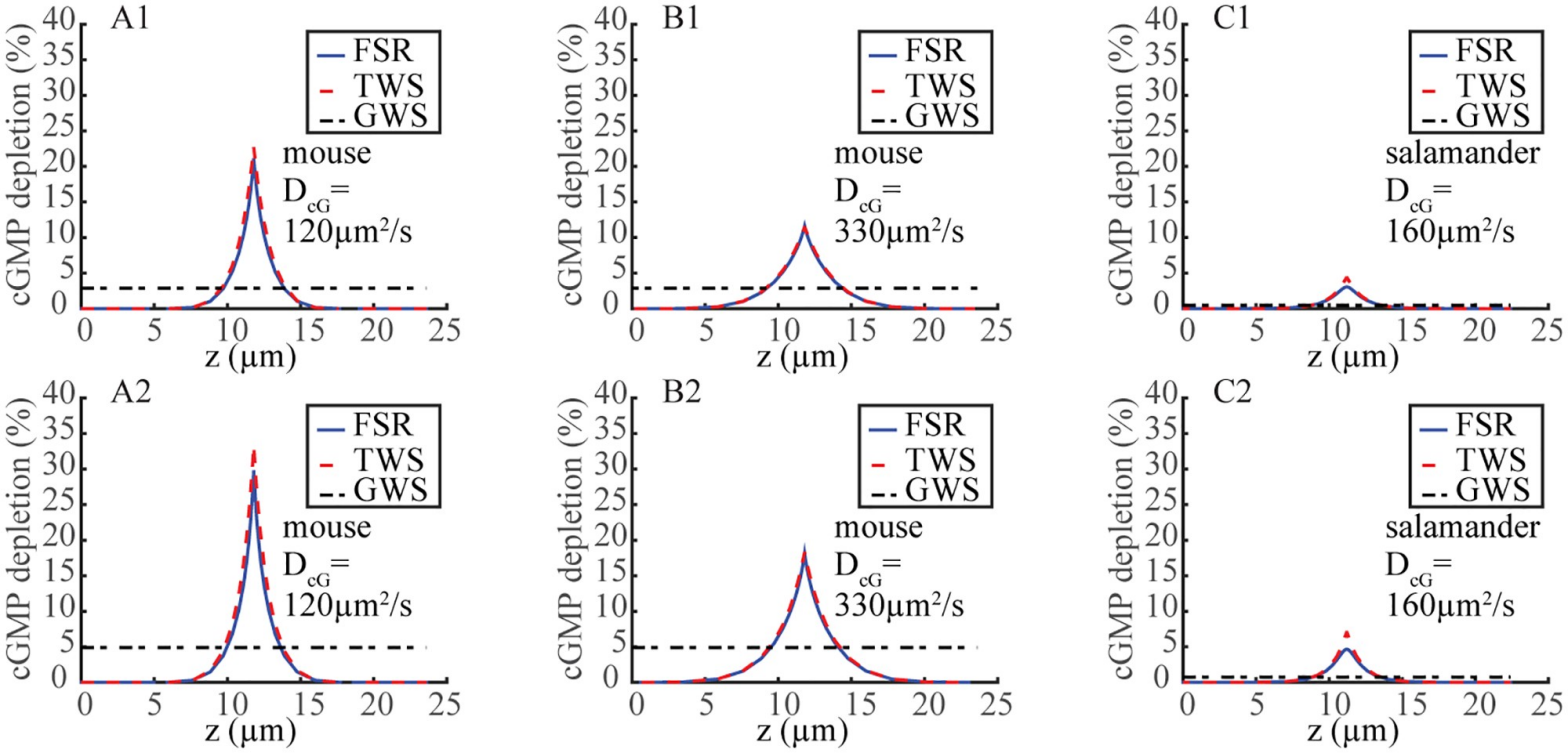
Axial spread of cGMP depletion at the t_peak_ of the SPR. Axial *z*-profiles of $z\to G(\rho ,\theta ,z,{\text{t}}_{\text{peak}})$ as defined in Eq 16, at the lateral boundary *ρ* = *R* of the ROS at time *t* = t_peak_. Deterministic deactivation of R* with lifetime *τ*_av_ = 0.11 s (**A1, B1**) or 2*τ*_av_ = 0.22 s (**A2, B2**) for mouse and *τ*_av_ = 0.625 s (**C1**) or 2*τ* = 1.25 s (**C2**) respectively for salamander.

Here (*ρ*, *θ*) are the polar coordinates on the activated disc, and *z* ∈ (0, *H*) is the longitudinal variable along the axis of the ROS. The locality of E* and that of Ca^2+^ feedback onto GC produce strong cGMP concentration gradients, both across the activated disc, and along the axis of the ROS. Fig 2 illustrates the time dynamics of G(ρ,θ,z,t) at the activation site (*ρ* = 0) and at the rim (*ρ* = *R*) of the activated disc as described by the FSR, TWS and GWS models. While mitigated by large diffusivities, which tend to equilibrate the [cGMP], the gradient between activation site and rim persists at all diffusion coefficients 0 < D_cG_ < ∞, and across species. The TWS and GWS models do not distinguish between center (*ρ* = 0) of the activated disc and its rim (*ρ* = *R*), thereby missing the dramatic drop in cGMP near the activation site. The magnitude of change is smaller with the GWS model because the cGMP reduction is distributed along the entire length of the ROS. The average axial *z*-profile of [cGMP](*ρ*, *θ*, *z*, t_peak_) depletion at the outer shell of the ROS at the peak of the SPR is shown in Fig 3. These profiles result from tracking locally, the movement of the second messengers within the ROS by the FSR model. The TWS produced a similar z-profile of [cGMP] drop that slightly overestimated the peak suppression. In marked contrast, the GWS model yielded a lower maximal [cGMP] depletion, spread uniformly along the ROS. Deterministic simulations were carried out for short and long lived R* with lifetime *τ*_av_ and 2*τ*_av_, where *τ*_av_ was computed as the sum of the means of the exponentially distributed random sojourn times *τ*_*j*_ assuming all phosphorylation steps have been taken ([12] Eqs 9–11). For mouse, by the parameters of B.1.1 of S1 Appendix and the sequence {*τ*_*j*_} introduced in ([12]) we computed *τ*_av_ = 0.11. For salamander, from the values of B.2.1 of S1 Appendix we computed *τ*_av_ ≈ 0.625s. Simulations were effected for FSR, TWS and GWS models. In all cases, the smallness of the peak relative [cGMP] depletion *at the outer shell* might suggest linearizing the expression of J_cG_ in Eq 2 about [cGMP] ≈ [cGMP]_dark_ ([15]), formula 11). Taylor expansion about this value yields

$$\begin{array}{c} 1-\frac{{\text{J}}_{\text{cG};\text{lin}}}{{\text{J}}_{\text{cG};\text{dark}}}\approx {\text{m}}_{\text{cG}}\frac{{\text{K}}_{\text{cG}}^{{\text{m}}_{\text{cG}}}}{{\text{K}}_{\text{cG}}^{{\text{m}}_{\text{cG}}}+[\text{cGMP}{]}_{\text{dark}}^{{\text{m}}_{\text{cG}}}}(1-\frac{\left[\text{cGMP}\right]}{{\left[\text{cGMP}\right]}_{\text{dark}}}). \end{array}$$ (17)

By this formula, the variability of the cGMP component of the current suppression can be computed from the variability of [cGMP] suppression. The process can be completed by introducing functions and quantities J_lin_, J_tot;lin_, j_tot;lin_, I_lin_(*t*), I_int;lin_ and I_area;lin_ as in Eqs 3–5 with J_cG;lin_ replacing J_cG_, and by estimating the variability of the SPR by computing the CV of these linearized functions. The Ca^2+^ component J_ex_ of the current was kept nonlinearized as in Eq 1, to stress that linearizing even a single component of the current generates distorsions in the response and variability. Thus the “linearized” quantities J_lin_, J_tot;lin_, j_tot;lin_, I_lin_(*t*), I_int;lin_ and I_area;lin_ are computed by using the linearization formula Eq 17 for the cGMP component of the current and the nonlinear relation Eq 1 for J_ex_.

#### Analyzing variability by stochastic simulations: Linearization and local cGMP depletion

A standard numerical WT experiment consisted in selecting *n* sojourn times *s*_*j*_ according to their exponential distributions with means *τ*_*j*_, putting them in Eq 13, from which, by a finite elements, Matlab based code ([32]), [E*]_*σ*_ was computed and fed into Eq 9, and its counterpart for [Ca^2+^], to compute [cGMP] and [Ca^2+^] as functions of space and time, at the lateral boundary of the ROS. Finally, using these [cGMP] and [Ca^2+^] values, current and current-functionals were computed from Eqs 1–5. It should be stressed that in Eqs 1 and 2, and hence the subsequent equations, only the boundary values of [cGMP] and [Ca^2+^] were used, since the lateral boundary of the ROS is where the current is generated. The process was repeated 1,000 times and from these outputs CV was computed for various ${\text{E}}_{\text{int}}^{*}$, I_int_, I and j_tot_, and their linearized counterparts. Simulations were carried out for the values of *volumic* diffusion coefficient D_cG_ = 120 *μ*m^2^s^−1^ ([13]) and D_cG_ = 330 *μ*m^2^s^−1^ ([15, 16]).

A set of virtual KO simulations, whose results are labelled by LIN (Linearization), was effected by the same steps and procedures where now the cGMP and Ca^2+^ fluxes Eqs 10 and 12 where linearized. A second set of virtual KO simulations, labelled NLD (Non Local Depletion), was effected by eliminating the local cGMP depletion by E* during deactivation, i.e., by keeping [cGMP] equal to its dark value [cGMP]_dark_ in the last term of Eq 9. A combination of these virtual KOs denoted by LIN+NLD enforces both effects.

Computations were then repeated using the TWS and GWS models to extract the implications to variability of various degrees of space resolution.

## Results and discussion

### Localization, nonlinearity and variability

Locality and nonlinearity inform variability at multiple levels. First, variability of E* passes to cGMP by means of the last term on the right-hand side of Eq 9. Second, variability of cGMP depletion inherited from E* gets deamplified by the nonlinear dependence of ion channel activity on [cGMP], by Eq 9. Between these two steps is the redistribution of cGMP, as it diffuses centrally from the outer shell, dampening the randomness of cGMP at the outer shell. Channel closure leads to a local decrease in [Ca^2+^], causing GC to replenish the cGMP at the outer shell, further dampening the randomness of cGMP there.

A first approximation of variability can be gained by comparing SPR simulations for deterministic short- and long-lived R*, e.g., deactivation with lifetimes of *τ*_av_ and 2*τ*_av_, as indicated in §. The relative difference of the outputs, Δ%, can be taken as a first variability estimator. For I_int_(*t*) and I_int;lin_(*t*) these values were computed at time *t* = t_peak_. The results are summarized in Tables 1 and 2 for mouse and Tables 3 and 4 for salamander. In all cases, Δ% of the functionals ${\text{E}}_{\text{max}}^{*}$ and ${\text{E}}_{\text{area}}^{*}$ exceeded that of the corresponding functionals I(t_peak_) and I_lin_(t_peak_) reflecting the incomplete transfer of variability from E* to I(⋅), as explained above.

**Table 1:** Mouse: First estimations of SPR variability and the errors introduced by linearization and loss of spatial resolution. Deterministic simulations with R* lifetimes *τ*_av_ and 2*τ*_av_, using the FSR, TWS and GWS models. Δ% is the relative difference of the outputs for each pair of runs. Computation of ${\text{E}}_{\text{area}}^{*}$, and the maximum value ${\text{E}}_{\text{max}}^{*}$ of E*(*t*), as defined by Eqs 14 and 15, for each of these two runs. Computation of I(t_peak_) using the nonlinear relations Eqs 1 and 2 and I_lin_(t_peak_) using the linearization Eq 17 for the cGMP component of the current and the nonlinear relation Eq 2 for J_ex_. ${\text{E}}_{\text{max}}^{*}$, ${\text{E}}_{\text{area}}^{*}$ and $G_{S}\left({\text{t}}_{\text{peak}}\right)$ are independent of the linearization Eq 17.

	FSR model, D_cG_ = 120 *μ*m^2^/s						
	${\text{E}}_{\text{max}}^{*}$	${\text{E}}_{\text{area}}^{*}\left(\text{s}\right)$	$G_{S}\left({\text{t}}_{\text{peak}}\right)\%$	I(t_peak_)%	I_lin_(t_peak_)%	I_area_(s)	I_area;lin_(s)
*τ*_av_	9.51	2.112	1.799	5.49	6.22	1.556	1.687
2*τ*_av_	15.24	4.266	2.430	7.09	8.40	2.448	2.735
Δ%	60.2	102.0	35.1	29.1	35.0	57.3	62.1
	FSR model, D_cG_ = 330 *μ*m^2^/s						
*τ*_av_	6.83	1.517	1.722	5.54	5.96	1.466	1.536
2*τ*_av_	10.95	3.063	2.612	8.12	9.04	2.522	2.707
Δ%	60.3	101.9	51.7	46.6	51.7	72.0	76.2
	TWS model, D_cG_ = 120 *μ*m^2^/s						
*τ*_av_	9.51	2.112	2.057	6.14	7.11	1.692	1.854
2*τ*_av_	15.24	4.266	2.878	8.10	9.94	2.712	3.089
Δ%	60.2	102.0	39.9	31.9	39.8	60.3	66.6
	TWS model, D_cG_ = 330 *μ*m^2^/s						
*τ*_av_	6.83	1.517	1.783	5.72	6.17	1.514	1.588
2*τ*_av_	10.95	3.063	2.756	8.51	9.54	2.634	2.838
Δ%	60.3	101.9	54.6	48.8	54.6	74.0	78.7
	GWS model						
*τ*_av_	9.51	2.112	2.857	9.56	9.90	2.446	2.502
2*τ*_av_	15.24	4.266	4.874	15.91	16.89	4.614	4.796
Δ%	60.2	102.0	70.6	66.4	70.6	88.6	91.7

**Table 2:** Mouse: *δ*% is the relative error between current density suppression J(t_peak_) at peak time t_peak_ and its linearized version J_lin_(t_peak_) for R* lifetimes *τ*_av_ and 2*τ*_av_, using the FSR, TWS and GWS model. **A**: Current density J(t_peak_) and J_lin_(t_peak_) computed at the rim of the activated disc; **B**: Total current suppression integrated over the outer shell I(t_peak_) and I_lin_(t_peak_).

	FSR				TWS				GWS	
	D_cG_ = 120 *μ*m^2^/s		D_cG_ = 330 *μ*m/s		D_cG_ = 120 *μ*m^2^/s		D_cG_ = 330 *μ*m^2^/s			
	*τ*_av_	2*τ*_av_	*τ*_av_	2*τ*_av_	*τ*_av_	2*τ*_av_	*τ*_av_	2*τ*_av_	*τ*_av_	2*τ*_av_
*δ*% A	30.2	45.6	15.4	25.2	32.9	52.3	15.2	25.6	3.5	6.1
*δ*% B	13.4	18.5	7.4	11.3	15.7	22.7	7.8	12.1	3.5	6.1

**Table 3:** Salamander: First estimations of SPR variability and the errors introduced by linearization and loss of spatial resolution. Deterministic simulations with R* lifetimes *τ*_av_ and 2*τ*_av_, using the FSR, TWS and GWS models. Δ% is relative difference of the outputs for each pair of runs. Computation of ${\text{E}}_{\text{area}}^{*}$, and the maximum value ${\text{E}}_{\text{max}}^{*}$ of E*(*t*) as defined by Eqs 14 and 15 for each of these two runs. Computation of I(t_peak_) using the nonlinear relations of Eqs 1 and 2 and I_lin_(t_peak_) using the linearization Eq 17 for the cGMP component of the current and the nonlinear relation Eq 2 for J_ex_. ${\text{E}}_{\text{max}}^{*}$, ${\text{E}}_{\text{area}}^{*}$ and $G_{S}\left({\text{t}}_{\text{peak}}\right)$ are independent of the linearization Eq 17.

	FSR model						
	${\text{E}}_{\text{max}}^{*}$	${\text{E}}_{\text{area}}^{*}\left(\text{s}\right)$	$G_{S}\left({\text{t}}_{\text{peak}}\right)\%$	I(t_peak_)%	I_lin_(t_peak_)%	I_area_(s)	I_area;lin_(s)
*τ*_av_	37.4	65.6	0.373	0.89	0.90	1.184	1.195
2*τ*_av_	63.9	127.3	0.555	1.32	1.35	2.097	2.128
Δ%	70.9	94.0	48.8	48.3	50.0	77.1	78.1
	TWS model						
*τ*_av_	37.4	65.6	0.422	1.00	1.01	1.301	1.315
2*τ*_av_	63.9	127.3	0.655	1.54	1.58	2.40	2.443
Δ%	70.9	94.0	55.2	54	56.4	84.5	85.8
	GWS model						
*τ*_av_	37.4	65.6	0.448	1.07	1.08	1.392	1.394
2*τ*_av_	63.9	127.3	0.728	1.75	1.76	2.685	2.694
Δ%	70.0	94.0	62.5	63.6	63.0	92.9	93.3

**Table 4:** Salamander: *δ*% is the relative error between current density suppression J(t_peak_) at peak time t_peak_ and its linearized version J_lin_(t_peak_) for R* average lifetimes *τ*_av_ and 2*τ*_av_, using the FSR, TWS and GWS models. **A**: Current density J(t_peak_) and J_lin_(t_peak_) computed at the rim of disc containing R*; **B**: Total current suppression integrated over the outer shell I(t_peak_) and I_lin_(t_peak_).

	FSR		TWS		GWS	
	*τ*_av_	2*τ*_av_	*τ*_av_	2*τ*_av_	*τ*_av_	2*τ*_av_
*δ*% A	2.13	3.29	3.18	5.19	0.31	0.50
*δ*% B	1.24	1.89	1.60	2.54	0.31	0.50

### Mouse dynamics: Linearization and variability

**Fig 4:**
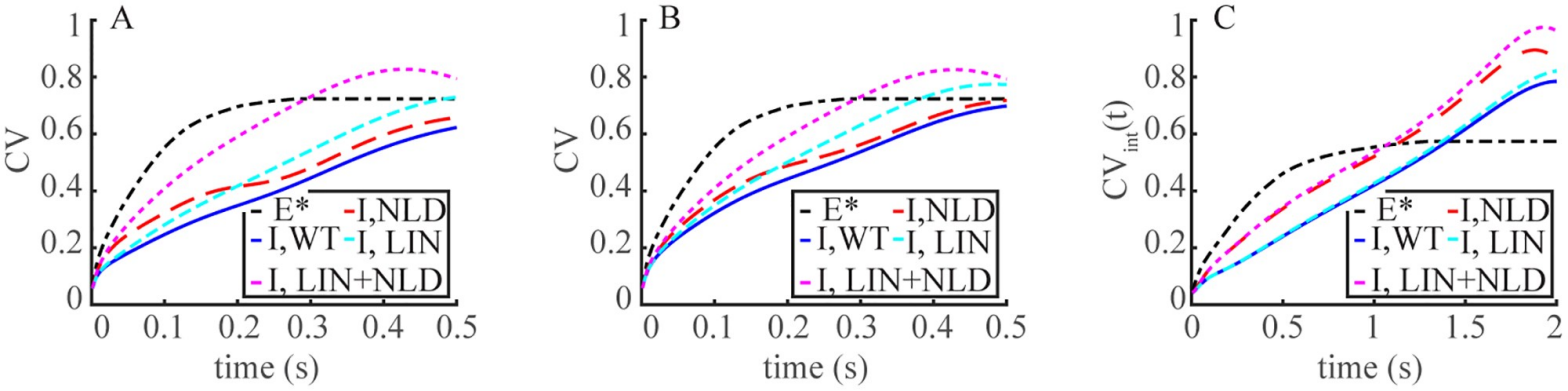
Overestimate of the variability of the SPR by linearization, disregarding the local cGMP depletion or suppressing spatial resolution of the ROS. Time dynamics of $t\to \text{CV}[{\text{E}}_{\text{int}}^{*}\left(t\right)]$ and *t* → CV(I_int_(*t*)), as defined in (Eqs 15 and 5). Computations are for WT (blue traces); NLD (non local depletion) (dashed red traces), i.e., with the coefficient of [E*]_*σ*_ in the right hand side of Eq 9 kept at its dark value [cGMP]_dark_; LIN (linearized) (dotted cyan), where the nonlinear fluxes of Eqs 10–12 were linearized; LIN+NLD (dotted purple), where both effects were enforced. **Panels A**: FSR Model; **Panels B**: TWS Model; **Panels C**: GWS Model. **Upper Panels (A1,B1,C1)**: Mouse with D_cG_ = 120 *μ*m^2^/s; **Middle Panels (A2,B2,C2)**: Mouse with D_cG_ = 330 *μ*m^2^/s; **Lower Panels (A3,B3,C3)**: Salamander with D_cG_ = 160 *μ*m^2^/s.

Tables 1 and 2 shows the relative errors in passing from the nonlinear relation Eq 2 to the linear relation Eq 17. These errors were in all cases at least of the order of 10% and were dramatically larger with lower diffusivity (D_cG_) and longer R* lifetime, casting doubt on the validity of the linearization Eq 17. Essentially, the computed SPR amplitude is larger with linearization and the discrepancy increases with the extent of cGMP depletion. Consequently, SPR variability was overestimated by linearizing the relation between current and [cGMP] and/or by increasing the diffusion coefficient D_cG_ and/or by by progressively disregarding the spatial resolution of the ROS, from FSR to GWS. These trends were explored further and validated by more rigorous determinations of CV. As was suggested by deterministic simulations, the distortions in computing the current suppression I_int_(*t*), by its nonlinear form Eq 2 and its linearized form I_int;lin_(*t*) as given in Eq 17 increased with longer R* lifetimes, hence they gave rise to errors in variability. While mitigated by averages, these errors persisted in the computation of the CV of current suppression. Fig 4 traces the dynamics of *t* → CV[I_int_(*t*)] for mouse rods, both for D_cG_ = 120 *μ*m^2^/s (Upper Panels) and D_cG_ = 330 *μ*m^2^/s (Middle Panels). Increased diffusivity raised variability. Linearizing the fluxes Eqs 10–12 also increased variability. Although the deterministic simulations suggested that the effect of linearization on I(t_peak_) variability was reduced with higher diffusivity (Table 1), computation of CV shows instead, that the relative error was substantial at all time points including t_peak_.

**Fig 5:**
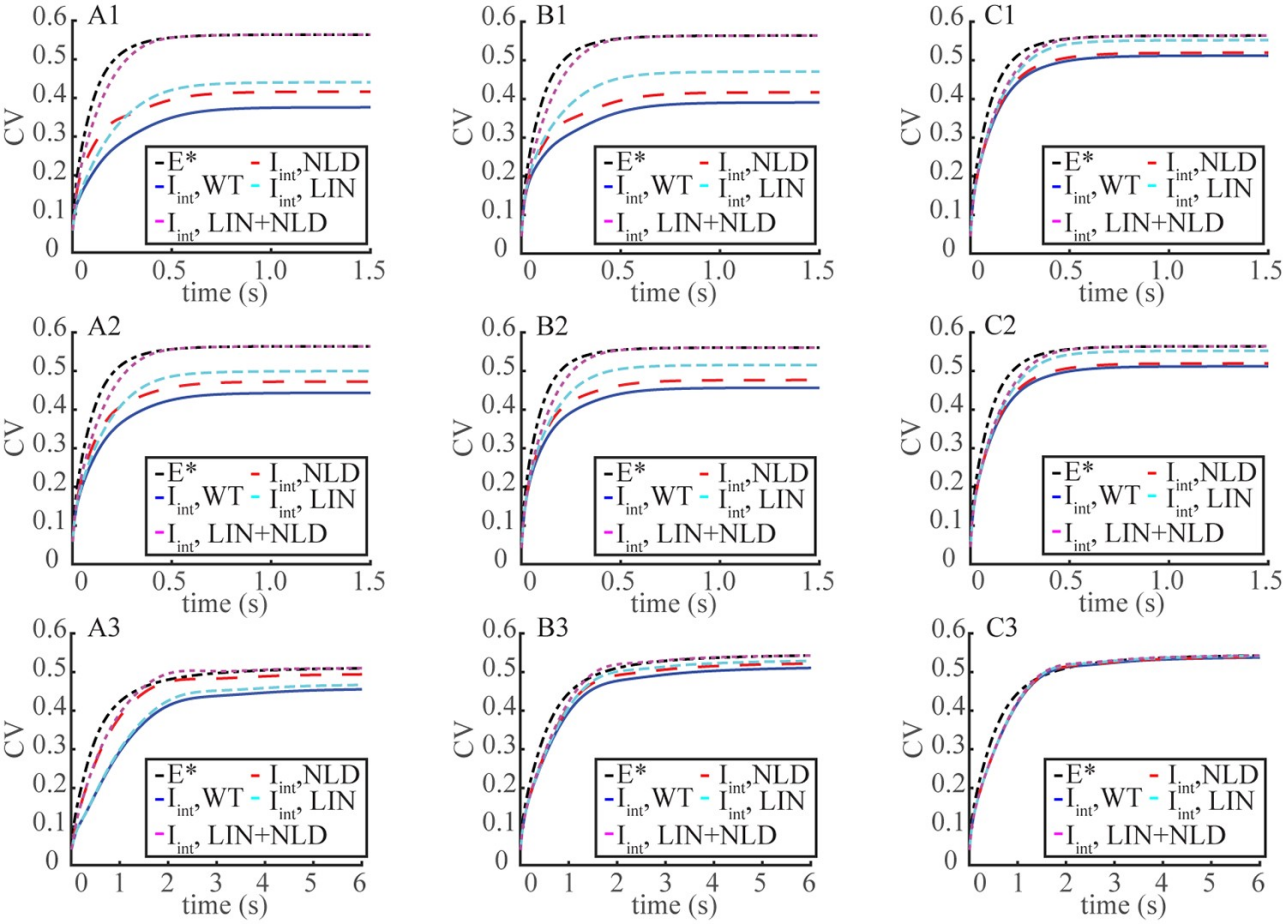
Overestimate of the variability of the SPR by linearization, disregarding the local cGMP depletion or suppressing spatial resolution of the ROS. Time dynamics of *t* → CV(E*(*t*)) and *t* → CV(I(*t*)), for WT (blue traces); NLD (non local depletion) (red traces), i.e., where the coefficient of [E*]_*σ*_ in the right hand side of Eq 9 was kept at its dark value [cGMP]_dark_; LIN (cyan traces), where the [cGMP] and Ca^2+^ fluxes of Eqs 10–12 were linearized; LIN+NLD (purple traces) where both effects were enforced. In all cases the FSR model was been used. **A**: Mouse with D_cG_ = 120 *μ*m^2^/s; **B**: Mouse with D_cG_ = 330 *μ*m^2^/s; **C**: Salamander. In all cases, keeping [cGMP] = [cGMP]_dark_ in Eq 9 and linearizing the nonlinear fluxes of Eqs 10–12 increased the variability of the SPR. For mouse, such an effect was less visible by increasing the diffusion coefficient D_cG_ from 120 *μ*m^2^/s to 330 *μ*m^2^/s, confirming that as D_cG_ → ∞ the system tends to a GWS model.

The approximation [cGMP] ≈ [cGMP]_dark_ in the last term on the right-hand side of Eq 9, overestimates the variability of the SPR, by introducing stronger sources of randomness in the cGMP cascade. This term is the mechanism by which E* passes along its variability to cGMP. If [cGMP] is locally depleted, near the random path *t* → **x**(*t*) of R*, the random contribution of [E*]_*σ*_ is mitigated by the local smallness of [cGMP], through the product [E*]_*σ*_[cGMP] in Eq 9, lowering the CV of the photocurrent. If, however, [cGMP] were to be uniformly close to its dark value [cGMP]_dark_ on the whole ROS and kept constant, the randomness of E* passes along to cGMP, and hence to the current suppression, linearly. Such an effect is local in nature, hence we refer to it as the *local depletion* of cGMP. The variability transferred from E* to cGMP is highest along the Brownian path of R*, where it is immediately reduced by a local sharp drop of cGMP (the coefficient of [E*]_*σ*_ in Eq 9), and progressively mitigated as [cGMP] migrates, and [E*]_*σ*_ terminates. Fig 5 shows that irrespective of the values of D_cG_, approximating [cGMP] ≈ [cGMP]_dark_ in the variability source term [E*]_*σ*_[cGMP] in Eq 9, (NLD) yields a larger *t* → CV(I(*t*)), particularly near the peak of the SPR, when cGMP depletion would be maximal. Finally if both local depletion and nonlinear effects are removed (LIN+LND), then the variability of the current suppression, after an initial delay due to diffusion eventually surpasses that of E*.

The deterministic simulations indicated that linearization increased variability as assessed by I_area_. Fig 4 extends the analysis of CV to *t* → CV(I_int_(*t*) dynamics for the FSR, TWS and GWS models. In all cases variability increased with decreased space resolution. In particular the GWS model passes along the variability of E* one-to-one to that of the response. In all cases by keeping [cGMP] = [cGMP]_dark_ in Eq 9 and linearizing the nonlinear fluxes of Eqs 10–12, or both, increased the variability of the SPR. With the combined effect, CV[I_int_(*t*)] matched CV[E*(*t*)] after a delay and since cGMP diffusion was no longer relevant, it was the same for both values of D_cG_ and for all models. While mitigated by an increasing degree of space resolution from FSR to GWS, localization and nonlinearity then appear as stabilizing factors to the randomness of the response.

Simulations of ([15]), with a TWS model, a deterministic single-step deactivation, and a relatively high longitudinal diffusion coefficient that favors rapid equilibration (D_cG_ ≈ 40 *μ*m^2^/s), give a small relative [cGMP] suppression and *z*-profiles comparable to Fig 3 **Upper panels**. It is inferred in ([15]) that because of the smallness of this [cGMP] drop, the rate equations can be linearized, the cGMP drop passes one-to-one to current suppression, and that, while no variability simulations were presented, the local cGMP depletion does not contribute to variability suppression. The simulations and arguments presented here disprove these conclusions (Fig 4). Such effects evidenced by the global/integral functional *t* → I_int_(*t*) persist when tracing the CV of the local in time current drop *t* → I(*t*), as reported in Fig 5. In all cases the variability suppression seems to act right from initial times and persists up to its own asymptotic limit. The use of these time-dependent functionals and their corresponding time-dependent variability is one of the novel points of this investigation with respect to those of ([12, 13]).

### Salamander dynamics: Linearization and variability

SPR kinetics are slower in salamander than in mouse in part because *ν*_RG_ activity is lower (≈ 185/s versus ≈ 330/s for mouse), R* deactivation is 5 times slower (*τ*_av_ ≈ 0.4 s versus *τ*_av_ ≈ 0.08 s for mouse) and cGMP has to travel farther from the activation site to the outer shell plasma membrane (radius *R* ≈ 5.5 *μ*m versus *R* ≈ 0.7 *μ*m for mouse). The relative [cGMP] suppression at the activation site *ρ* = 0, is comparable to mouse and it remains large for longer times (Fig 2). Such a dramatic local drop is recorded by the FSR model and not detected by the TWS and GWS models. As expected, the cGMP suppression at the rim is much smaller in salamander than in mouse, with the TWS model overestimating it. The *z*-profiles of $G(R,\theta ,z,{\text{t}}_{\text{peak}})$, i.e., the *S*-integrated relative cGMP suppression, are much smaller than in mouse (Fig 3), suggesting a linearization of the form Eq 17 might be valid for salamander. Table 3 computes the variability of the functionals I(t_peak_), and I_area_ from the nonlinear relation Eq 2 and their counterparts I_lin_(t_peak_) and I_area;lin_ from the linearized Eq 17, using the FSR, TWS and GWS models, for deterministic simulations with R* lifetimes *τ*_av_ and 2*τ*_av_. The functionals ${\text{E}}_{\text{max}}^{*}$, ${\text{E}}_{\text{area}}^{*}$ and $G_{S}\left({\text{t}}_{\text{peak}}\right)$ are independent of the linearization. The distortions introduced by linearization were considerably smaller in salamander than in mouse. The relative errors between I(t_peak_) and I_lin_(t_peak_) for the same deterministic runs did not exceed 3.11% for the FSR model and 5% for the TWS model, although the cumulative error over time with I_area_ was larger, of the order of 11%. Similarly, the effect of decreasing spatial resolution was somewhat less pronounced than in mouse. Thus in random trials with randomly long- and short-lived R*, the errors due to linearization were not expected to increase the coefficient of variation of the current suppression as much as in mouse. Simulations with stochastic R* shutoff substantiate these predictions (lower panels of Fig 4). For the FSR model, linearization of the J_cG_-cGMP relation Eq 2 increases the CV of the current suppression only slightly, whereas the increase is more significant for the TWS model. Roughly speaking, in the TWS model, the variability of cGMP, inherited from E*, is passed along to I_int_(*t*) with minor suppression. The variability of E* however is significantly suppressed as it is transmitted to cGMP because of the depletion term [E*]_*σ*_[cGMP] in Eq 9. If [cGMP] were to be approximated to [cGMP]_dark_, the coefficient of [E*]_*σ*_ would be relatively large and constant, and hence the randomness of E* would be transmitted one-to-one to cGMP, and passed to I_int_(*t*) essentially unchanged. The sharp local drop of cGMP (Fig 2), makes the coefficient of [E*]_*σ*_ relatively small, resulting in a lower transmission of variability from E* to cGMP. The latter is further reduced by its migration by diffusion to the outer shell, and ultimately transmitted to the current drop. Hence, this local depletion, present in mouse and salamander, emerges as a key variability suppression mechanism.

### The effects of Ca^2+^ feedback on variability

**Fig 6:**
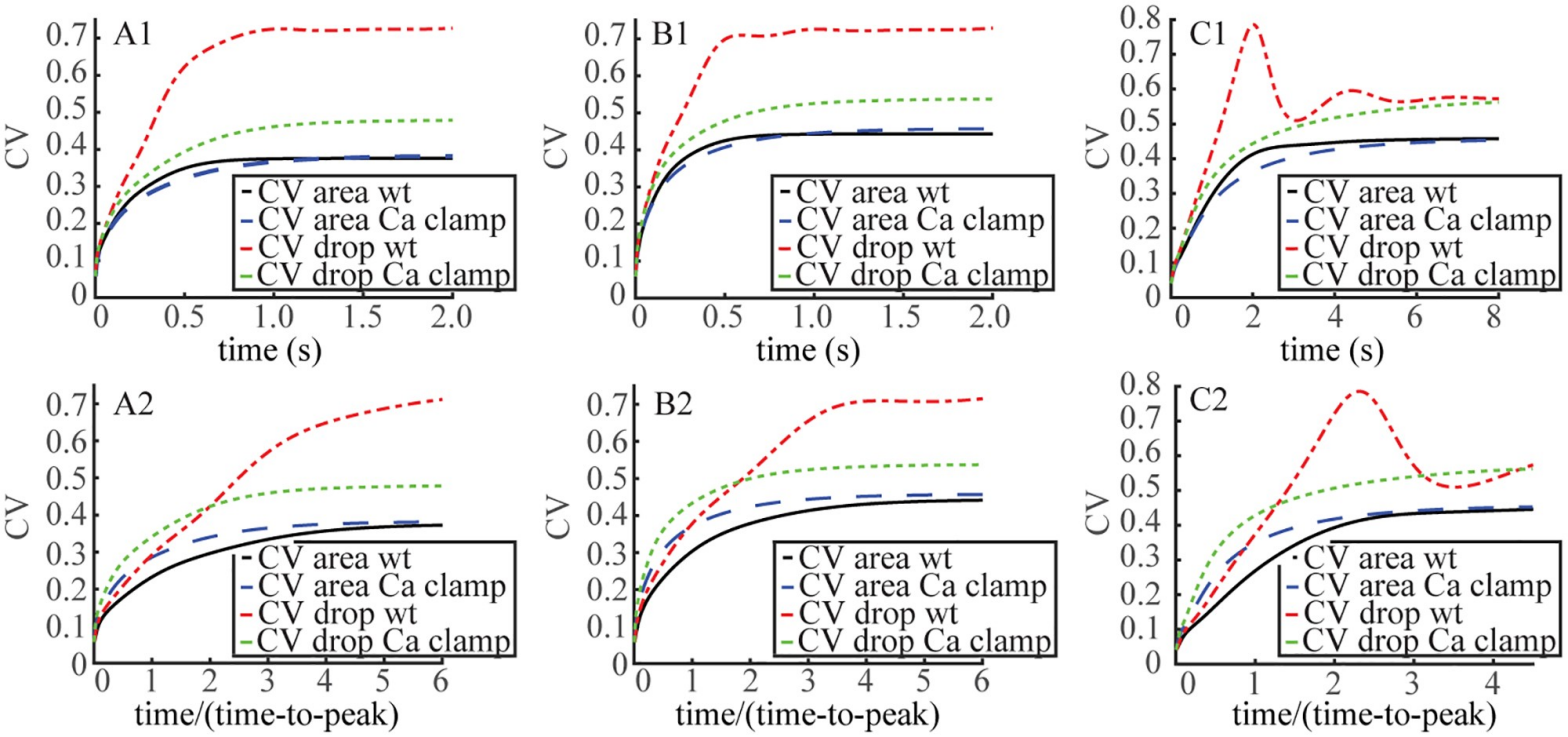
Crossing of variability curves revealed by viewing the WT and Ca^2+^ clamped SPR on their intrinsic time scales. Time dynamics of CV(I_int_(⋅)), CV[I(⋅)] for WT, (black, red traces) and Ca^2+^ clamped conditions (dashed blue, green traces) for mouse (**A1,B1,A2,B2**) and salamander (**C1,C2**). D_cG_ was set to 120 *μ*m^2^/s (**A1, A2**) or to 330 *μ*m^2^/s (**B1, B2**). The functionals I_int_(*t*) and I(*t*) are defined in Eqs 3, 4 and 5. In all cases the FSR model was in force. **Upper Panels A1, B1, C1**: Time dynamics of *t* → CV(I_int_(*t*)) (CV-area), *t* → CV[I(*t*)] (CV-drop) in absolute times *t* in s. **Lower Panels, A2, B2, C2**: Time dynamics of *τ* → CV(I_int_(*τ*)) (CV-area), *τ* → CV[I(*τ*)] in relative rescaled times *τ* = *t*/t_peak_, so that *τ* = 1 corresponds, in each case, to the amplitude of the SPR.

Molecules of cGMP located distant from E*and those generated by GC during the course of the SPR roughly speaking, act as first responders that rush by diffusion to the activation site to replenish cGMP being randomly depleted of by E*. In doing so, they dissipate “globally” the randomness of E* on the activated disc, dampening the transfer of randomness to the nearest CNG channels at the outer shell by which variability in the current is generated. With Ca^2+^ feedback intact, GC is stimulated close to the CNG channels that are experiencing the greatest light-induced fall in [cGMP], to further dampen “locally” the transfer of randomness. With Ca^2+^ clamped, the GC mediated production of cGMP remains constant; local dampening is absent, leaving global dampening to act alone, thereby elevating the variability of the current. Experimental results of ([16, 33]) suggest that mutant mouse rods lacking GCAPs (GCAP^−^/^−^) essentially behave as if Ca^2+^ were clamped. CV[I(t_peak_)] increased to 0.42 from a baseline of 0.34 for WT ([16]). Simulations of ([16]) with a TWS model, using the same stochastic deactivation mechanism as in ([12, 13]), report an increase of CV[I(t_peak_)] to 0.38 for GCAP^−^/^−^ rod vs 0.32 for WT. These interpretations were extrapolated from Table F of Fig 6 of ([16]). As a one time point value, CV[I(t_peak_)] does not capture the variability over the time course of the SPR. According to an FSR model, clamping Ca^2+^ appears to have a negligible effect on CV of the cumulative/integral functional I_area_ ([12, 13]). The variability of I_area_ is considerably higher than the variability of I(t_peak_) ([11]), but as indicated above, the former compiles the fluctuations of I(*t*) over the entire time course of the response. We repeated the simulations of ([12, 13]) for several values of D_cG_, using FSR, TWS, and GWS models applied to salamander as well as mouse rods to see whether these results hold across species. We mapped the CV for several functionals including I(*t*) over the duration of the SPR and extended simulations to salamander.

The simulations are reported in Tables 5 and 6 and Fig 6. In all models and irrespective of D_cG_, clamping Ca^2+^ in Eq 10, i.e., removing the Ca^2+^ feedback, had no effect on the CV(I_area_), confirming the results of ([12, 13]). CV of the integral current suppression I_int_(*t*) for Ca^2+^-clamped was initially slightly lower than the corresponding one for WT, for later times was slightly higher, and asymptotically it was the same. Since I_area_ = lim_*t*→∞_ I_int_(*t*) integrates the variability over the entire time course of the SPR, the lower variability at short, initial times was compensated by a slightly higher one at longer times, thereby explaining why the CV(I_area_) did not detect a rise in variability due to clamping Ca^2+^. These conclusions are made possible by tracing the variability of the response by means of the time-dependent functional I_int_(*t*) as opposed to the time-global functional I_area_ as in ([12, 13]).

**Table 5:** Mouse. CV for I(t_peak_) and I_area_ by the FSR, TWS and GWS models, for D_cG_ = 120 *μ*m^2^/s and D_cG_ = 330 *μ*m^2^/s. CVs computed for the WT mouse and the following *virtual* conditions. **LIN**: i.e., the nonlinear fluxes Eqs 10–12 were linearized. **NLD (non-local depletion)**: i.e., in the product [E*]_*σ*_[cGMP] in the last term on the right hand side of Eq 9, [cGMP] were kept constant at its dark value. **LIN+NLD**: both effects are enforced. **Ca^2+^-clamp**: Ca^2+^ dependence of cyclase activity was removed, i.e., in the expression of *F* in Eq 10, [Ca^2+^] was kept at its dark value [Ca^2+^]_dark_. In all cases $\text{CV}({\text{E}}_{\text{max}}^{*})=0.45$ and $\text{CV}({\text{E}}_{\text{area}}^{*})=0.56$.

	CV[I(t_peak_)]	CV(I_area_)(s)
FSR	D_cG_ = 120 *μ*m^2^/s	
WT	0.29	0.38
LIN	0.36	0.44
NLD	0.37	0.42
LIN+NLD	0.52	0.56
Ca^2+^-clamp	0.34	0.38
FSR	D_cG_ = 330 *μ*m^2^/s	
WT	0.38	0.44
LIN	0.44	0.50
NLD	0.43	0.47
LIN+NLD	0.52	0.56
Ca^2+^-clamp	0.43	0.46
TWS	D_cG_ = 120 *μ*m^2^/s	
WT	0.32	0.39
LIN	0.40	0.47
NLD	0.37	0.42
LIN+NLD	0.52	0.56
Ca^2+^-clamp	0.36	0.40
TWS	D_cG_ = 330 *μ*m^2^/s	
WT	0.41	0.46
LIN	0.47	0.52
NLD	0.44	0.48
LIN+NLD	0.53	0.56
Ca^2+^-clamp	0.45	0.47
GWS	GWS	
WT	0.47	0.51
LIN	0.51	0.55
NLD	0.49	0.52
LIN+NLD	0.52	0.56
Ca^2+^-clamp	0.49	0.51

**Table 6:** Salamander. CV for I(t_peak_) and I_area_ by the FSR, TWS and GWS models. CVs computed for the WT and the following *virtual* conditions. **LIN**: i.e., the nonlinear fluxes Eqs 10–12 were linearized. **NLD (non-local depletion)**: in the product [E*]_*σ*_[cGMP] in the last term on the right hand side of Eq 9, [cGMP] was kept constant at its dark value. **LIN+NLD**: both effects were enforced. **Ca^2+^-clamp**: Ca^2+^ dependence of cyclase activity was removed, i.e., in the expression of *F* in Eq 10, [Ca^2+^] was kept at its dark value [Ca^2+^]_dark_. In all cases $\text{CV}({\text{E}}_{\text{max}}^{*})=0.44$ and $\text{CV}({\text{E}}_{\text{area}}^{*})=0.51$ for FSR and $\text{CV}({\text{E}}_{\text{max}}^{*})=0.47$ and $\text{CV}({\text{E}}_{\text{area}}^{*})=0.54$ for TWS and GWS. The CVs of E* for all models converge to identical values with larger sample number.

	CV[I(t_peak_)]	CV(I_area_)(s)
	FSR	
WT	0.37	0.46
LIN	0.38	0.47
NLD	0.45	0.50
LIN+NLD	0.46	0.51
Ca^2+^-clamp	0.43	0.45
	TWS	
WT	0.44	0.51
LIN	0.46	0.53
NLD	0.46	0.52
LIN+NLD	0.48	0.54
Ca^2+^-clamp	0.49	0.51
	GWS	
WT	0.47	0.54
LIN	0.48	0.54
NLD	0.47	0.54
LIN+NLD	0.48	0.54
Ca^2+^-clamp	0.53	0.54

Early during the rising phase of the SPR, CV of the “pointwise” current suppression I(*t*) for Ca^2+^-clamped lay below the corresponding CV for WT, irrespective of diffusion coefficient and across species (Fig 6, Upper panels), seemingly at odds with the values of Tables 5 and 6. The reason is that the SPR for WT and Ca^2+^-clamped peak at different times. In recordings of “Ca^2+^-clamped” GCAP^−^/^−^ mouse rods, the SPR reaches its maximum at a t_peak_ 2-3 fold later than in WT, and rises to an amplitude ≈ 4 fold higher than that of WT ([33–35]). As a consequence the CV of I(t_peak_) for WT and Ca^2+^-clamped are comparable only in their own intrinsic time scales *τ* = *t*/t_peak_.

Fig 6 Lower Panels follow the dynamics of the CV of the pointwise functionals *τ* → I_int_(*τ*) and *τ* → I(*τ*), along the relative dimensionless time for mouse [Lower Panels A (D_cG_ = 120 *μ*m^2^/s) and B (D_cG_ = 330 *μ*m^2^/s)], and salamander [Lower Panel C]. A “crossing of variability curves” while invisible for CV[I_int_(*t*)] in absolute times (Fig 6, Upper Panels), was revealed in relative times (Fig 6, Lower Panels). Precisely, clamping Ca^2+^ augmented the variability of the SPR with respect to WT, for a time course up to roughly 2 fold longer than its peak time for mouse, and roughly 1.3 fold longer than its time to peak for salamander. The dynamics of CV[I_int_(⋅)] for Ca^2+^-clamped remained above that of WT at all relative intrinsic times. For this functional crossing of the variability curves disappeared on the relative time scale.

**Fig 7:**
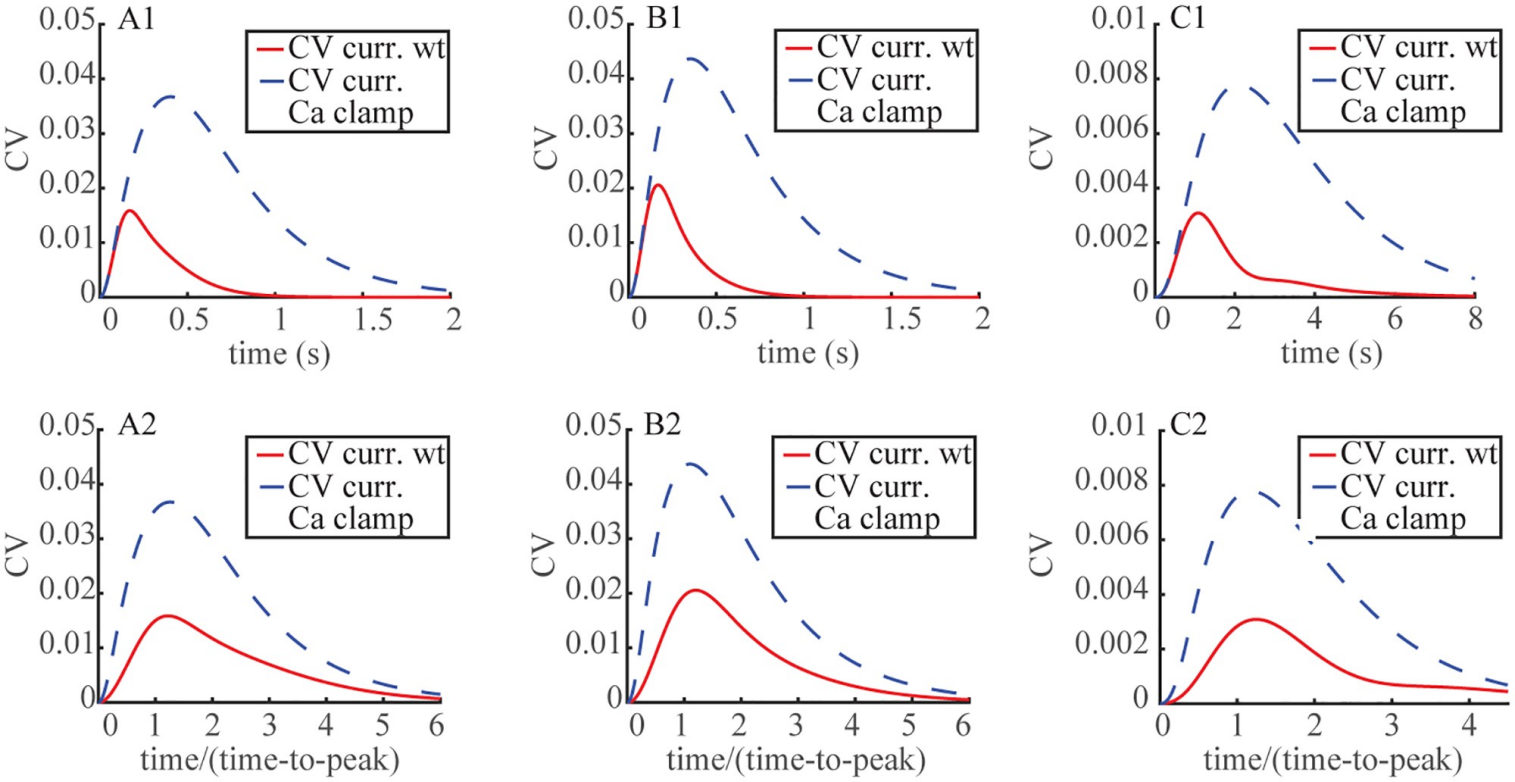
Increased SPR variability with Ca^2+^ clamping, as assessed with the j_tot_(·) functional defined in Eq 3. Time dynamics of CV(j_tot_(·)) (current) are shown for WT (red traces) and Ca^2+^ clamped (dashed blue traces) SPRs. In all cases the FSR model was in force. **Panels A1, A2**: mouse with D_cG_ = 120 *μ*m^2^/s. **Panels B1, B2**: mouse with D_cG_ = 330 *μ*m^2^/s. **Panels C1, C2**: salamander. **Upper Panels A1, B1, C1**: Time dynamics of *t* → (j_tot_(*t*)) (current), in absolute times *t* in s. **Lower Panels A2, B2, C2**: Time dynamics of *τ* → (j_tot_(*τ*)) (current), in relative times *τ* = *t*/t_peak_, so that *τ* = 1 corresponds, in each case, to the amplitude of the SPR.

The “crossing of variability curves” is an epiphenomenon arising as an artifact of the choice of functionals by which one measures variability. In § we indicated that the CV of I(*t*) might be ill defined since the numerator of Eq 6 is the standard deviation of j_tot_, which, as such, needs to be normalized by the statistical mean of J_tot_(*t*) and not that of I(*t*). In formula Eq 6 the denominator is the statistical mean of the current drop. Now by the Ca^2+^-clamped recordings of ([33–35]) the SPR amplitude is ≈ 4 fold higher than that of WT. Assuming a comparable difference continues to hold along the time course of the SPR, the denominator in Eq 6 is considerably larger than the corresponding one for WT. This accounts for the drastic difference between the *t* → CV[I(*t*)] curve for WT and Ca^2+^-clamped. It also suggests measuring variability by the J_tot_(*t*) functional. Fig 7 traces the dynamics of *t* → CV[j_tot_(*t*)] as defined in Eq 7. By this measure the variability for Ca^2+^-clamped was at all times, absolute or relative, higher than that of WT. Thus the nonlinear and non-constant dependence on [Ca^2+^] of cyclase activity that leads to a light-induced up-regulation of cGMP synthesis as appearing in Eq 10, acted as a variability suppressor of the SPR.

It is worth stressing that the integral defining j_tot_(*t*) in Eq 3 is extended on the outer shell *S*, whereas variability originates elsewhere. For long times, channels on *S* near the activated level z_*_ keep closing and only relatively few are permitted to reopen due to lack of cGMP production by GC. Thus for long times, under Ca^2+^ clamped conditions, [cGMP] is small at *S*, and the randomly long lived R* generate smaller random fluctuations of cGMP on *S*, and hence by formula Eqs 2–5 smaller current fluctuations. The reduction in current is made more dramatic by the nonlinear relation Eq 2. Indeed as [cGMP] → 0 on *S*, the corresponding current J_cG_ goes to zero much faster, precisely as $[\text{cGMP}{]}^{{\text{m}}_{\text{cG}}}$ with m_cG_ ≈ 4. However, again by the limited cGMP production by GC, the system returns only very slowly to its steady state and hence the statistical average of j_tot_(*t*) is not yet close to j_dark_. These information in formula Eq 6 suggest a lowering of CV[I(*t*)] for times *t* > 2t_peak_ (Fig 6, Lower Panels).

In the WT situation, closed channels on *S* are continuously reopened by the input of cyclase mediated cGMP. As a consequence, randomly long lived R* cause continued random generation of cGMP on the outer shell and hence random current and current drop. However again by the Ca^2+^ stimulated continued cGMP production by GC, the system rapidly equilibrates and after a time *t* > 2t_peak_ the statistical average of j_tot_(*t*) is close to its dark value j_dark_, generating, by formula Eq 6, a large CV.

For this reason the curves of *t* → CV[I(*t*)] for WT and clamped Ca^2+^ in Fig 6 cross invert their variabilities at times larger than ≈ 1.5t_peak_. Fig 7 tracks the current and hence cGMP, as a direct consequence of the relation Eq 2 and shows that after roughly the same time threshold of ≈ 1.5t_peak_, the variability of j_tot_(*t*) decreases for clamped Ca^2+^ much faster than for WT. These results underscore that the notion of “variability” is not an absolute one and depends on the choice of functionals used to measure it, each extracting different information depending on time and location of the response.

## Conclusion

Animals have two different types of photoreceptors, ciliary (e.g., vertebrates) and rhabdomeric (e.g., insects) ([36, 37]). Some animals (e.g., marine rag-worm Platynereis) have both types ([38]). The phototransduction cascades in these two types of photoreceptors are quite different, yet one thing they share is multiple layers of photopigment-containing membranes. It is widely believed that this type of organization mostly serves to increase photon catch; the chance that the photon will be absorbed is greatly increased by having the light pass through many photopigment-containing membranes. In rhabdomeric and in ciliary cone photoreceptors, phototransduction occurs within the confines of restricted compartments to prevent second messengers from getting diluted in a large volume of cytoplasm. However, the design changed for ciliary rods for which there are multiple layers of cytoplasm partially separated by membranous discs containing R. Our modeling suggests a reason for this geometry.

The loose segmentation of the cytoplasm by multiple photopigment-containing membranes ensures that light-evoked changes in the concentrations of second messengers retain some locality. This appears to serve an important purpose. Change produced by the activation of just a few molecules of signal transducers and effectors in response to light capture by a single photopigment molecule are “diluted slightly” in a larger volume of photoreceptor cytoplasm. The limited dissipation of these changes suppresses the biochemical effect of inevitable random variation of the number of the effectors activated by a single photopigment, thereby reducing the variability of SPR.

Calcium clamp experiments show that if the calcium concentration is kept constant, the resulting variability at early time points is higher than in WT rods. Importantly, the variability is lower before the peak response is reached, i.e., at the time when bipolar cells respond to rod activation ([39]). Calcium clamping decreases the variability at later time points (Fig 6, Lower Panels). However, this late effect appears to be irrelevant for fully dark adapted bipolar cells, and therefore would not contribute to the reliability of vision. In summary, strict localization of both the cGMP response to a photon and calcium feedback, which is ensured by complex photoreceptor geometry with slivers of cytoplasm separated by discs forming diffusion barriers, serves to suppress SPR variability, which is inevitably generated by the randomly activated and deactivated biochemical cascade. Thus, by using photoreceptors with multi-disc structure nature apparently devised a way to overcome the limitations of biochemical reactions. Rods generate the response to single photons that is significantly less variable, and therefore more reliable, than pure biochemistry would allow. This feature is captured by FSR model, but missed by TWS and GWS models of phototransduction. For more information, see S1 Appendix.

## Supporting information

S1 Appendix Collected appendices A-D. Appendices include the weak formulations for 2nd messengers and the G-protein cascade (A), parameters used for mouse and salamander models (B), a calibration for the choice of *ν*_RG_ when D_cG_ = 330 *μ*m^2^/s (C), and a theoretical calculation linking volumic and longitudinal diffusivities (D).
(PDF)
