# Supplementary material for: Local, nonlinear effects of cGMP and Ca2+ reduce single photon response variability in retinal rods
Source: PLoS One. 2019 Dec 5;14(12):e0225948. doi: 10.1371/journal.pone.0225948 (PMC6894879; doi:10.1371/journal.pone.0225948)
Supplement: S1 Appendix — Appendices include the weak formulations for 2nd messengers and the G-protein cascade (A), parameters used for mouse and salamander models (B), a calibration for the choice of νRG when DcG = 330 μm2/s (C), and a theoretical calculation linking volumic and longitudinal diffusivities (D). (PDF) [file pone.0225948.s001.pdf]

APPENDICES to Local, Nonlinear Effects of cGMP and Ca^{2+} Reduce Single Photon Response Variability in Retinal Rods

Giovanni Caruso
CNR, Ist. Tecnologie Applicate ai Beni Culturali
Via Salaria Km. 29,300- C.P. 10 Monterotondo St., Roma, Italy
email: giovanni.caruso@itabc.cnr.it

Vsevolod V. Gurevich
Department of Pharmacology, Vanderbilt Univ. Medical Center
2200 Pierce Avenue, Preston Res. Building, Rm 452
Nashville, TN 37232
email: Vsevolod.Gurevich@vanderbilt.edu

Colin Klaus
The Mathematical Biosciences Institute, The Ohio State University
1735 Neil Avenue, Columbus, OH 43210
email: klaus.68@mbi.osu.edu

Heidi E. Hamm
Department of Pharmacology, Vanderbilt Univ. Medical Center
2200 Pierce Avenue, Preston Res. Building, Rm 452
Nashville, TN 37232
email: heidi.hamm@vanderbilt.edu

Clint L. Makino*
Boston University School of Medicine
Department of Physiology and Biophysics
Ctr. for Advanced Biomedical Research, W402
700 Albany Street, Boston, MA 02118-2526
email: cmakino@bu.edu

Emmanuele DiBenedetto†
Department of Mathematics, Vanderbilt University
1326 Stevenson Center, Nashville, TN 37240
email: em.diben@vanderbilt.edu

*Co-corresponding author
†Corresponding author

A Appendix. Dynamics of the Cascade

A.1 Symbolism

$$
\begin{aligned}
H &= \text{height of the ROS}\\
R &= \text{radius of the discs inside the ROS (disregarding incisures)}\\
D_R &= \text{disc of radius } R \text{ centered at the origin of } \mathbb{R}^2\\
m &= \text{number of incisures}\\
\mathcal{V}_j &= \text{limiting j}^{\text{th}} \text{ incisures, assimilated to segments of length } R - r_{o,j}\\
r_j &= \text{radial variable on } \mathcal{V}_j \text{ with origin at } r_{o,j}\\
\theta_{\varepsilon_o,j}(r_j) &= \text{geometry of the j}^{\text{th}} \text{ incisure with tip at } r_{o,j}\\
D_{\text{eff}} &= D_R - \cup_{j=1}^m \mathcal{V}_j \;\; \text{effective domain of the activation cascade}\\
k &= \text{number of distinct activated discs each by a single photon}\\
D^*_{i,\text{eff}} &= \text{i}th \text{ activated disc}\\
\Omega &= D_R \times (0,H) \text{ limiting cylinder enclosing the stack of discs } D_R\\
\Omega_{\text{eff}} &= D_{\text{eff}} \times (0,H) \text{ limiting domain available for diffusion of cGMP and Ca}^{2+}\\
\mathcal{B}_j &= \mathcal{V}_j \times (0,H) \;\text{ limiting vertical rectangles cut on the limiting ROS}\\
&\quad\ \text{by the limiting incisures aligned in series}\\
S &= \text{limiting outer shell (same as lateral boundary of } \Omega)\\
dS &= \text{surface measure on } S\\
\varepsilon_o &= \text{width of each disc}\\
\nu\varepsilon_o &= \text{width of each interdiscal space}\\
\sigma\varepsilon_o &= \text{width of the outer shell}\\
1-\mu_o &= \text{volume ratio of cytosol to the volume of the ROS}\\
[\text{cGMP}] &= [\text{cGMP}] \text{ in the interior of the limiting ROS}\\
[\text{cGMP}]_* &= [\text{cGMP}] \text{ at the activated disc(s)}\\
[\text{cGMP}]_S &= [\text{cGMP}] \text{ in the limiting outer shell}\\
[\text{cGMP}]_{\mathcal{B}_j} &= [\text{cGMP}] \text{ on } \mathcal{B}_j\\
[\text{Ca}^{2+}] &= [\text{Ca}^{2+}] \text{ in the interior of the limiting ROS}\\
[\text{Ca}^{2+}]_* &= [\text{Ca}^{2+}] \text{ at the activated disc(s)}\\
[\text{Ca}^{2+}]_S &= [\text{Ca}^{2+}] \text{ in the limiting outer shell}\\
[\text{Ca}^{2+}]_{\mathcal{B}_j} &= [\text{Ca}^{2+}] \text{ on } \mathcal{B}_j\\
\nabla_S &= \text{gradient along the cylindrical variables of } S\\
\nabla_{\mathcal{B}_j} &= \text{gradient along the } (r_j,z) \text{ variables of } \mathcal{B}_j\\
\nabla_{(x,y)} &= \text{gradient along the horizontal variables } (x,y)
\end{aligned}
$$

A.2 Weak Formulation of the Dynamics of cGMP

$$
\begin{aligned}
&(1-\mu_o)\Big\{ \iiint_{\Omega_{\rm eff}} [\mathrm{cGMP}](t)\varphi(t)\,dxdydz - \iiint_{\Omega_{\rm eff}} [\mathrm{cGMP}]_{\rm dark}\varphi(0)\,dxdydz \\
&\qquad + \int_0^t \iiint_{\Omega_{\rm eff}} \big\{ -[\mathrm{cGMP}]\varphi_t + \mathrm{D}_{\rm cG}\nabla_{(x,y)}[\mathrm{cGMP}]\cdot\nabla_{(x,y)}\varphi \\
&\qquad\qquad - \big[\alpha([\mathrm{Ca}^{2+}]) - \beta_{\rm dark}[\mathrm{cGMP}]\big]\varphi\big\} dxdydz\,d\tau \Big\}_{\text{interior}} \\
&+\nu\varepsilon_o\Big\{ \sum_{i=1}^{k} \iint_{D^*_{i,\rm eff}} \big\{[\mathrm{cGMP}]_*(t)\varphi(t) - [\mathrm{cGMP}]_{\rm dark}\varphi(0)\big\} dxdy \\
&\qquad + \sum_{i=1}^{k} \int_0^t \iint_{D^*_{i,\rm eff}} \Big\{ -[\mathrm{cGMP}]_*\varphi_t + \mathrm{D}_{\rm cG}\nabla_{(x,y)}[\mathrm{cGMP}]_*\cdot\nabla_{(x,y)}\varphi \\
&\qquad - \Big(\alpha([\mathrm{Ca}^{2+}]_*) - \beta_{\rm dark}[\mathrm{cGMP}]_* - \frac{\mathrm{k}^*_{\sigma;\rm hyd}}{\nu\varepsilon_o}[\mathrm{E}^*]_\sigma[\mathrm{cGMP}]_*\Big)\varphi\Big\} dxdyd\tau \Big\}_{\substack{\text{activated}\\ \text{discs}}} \\
&+\sigma\varepsilon_o\Big\{ \iint_S \big\{[\mathrm{cGMP}]_S(t)\varphi(t) - [\mathrm{cGMP}]_{\rm dark}\varphi(0)\big\} dS \\
&\qquad + \int_0^t \iint_S \big\{ -[\mathrm{cGMP}]_S\varphi_t + \mathrm{D}_{\rm cG}\nabla_S[\mathrm{cGMP}]_S\cdot\nabla_S\varphi\big\} dSd\tau \Big\}_{\text{outer shell}} \\
&+2\Big\{ \sum_{j=1}^{m} \iint_{\mathcal{B}_j} r_j\theta_{j,\varepsilon_o}(r_j)\big\{[\mathrm{cGMP}]_{\mathcal{B}_j}(t)\varphi(t)dr_jdz - [\mathrm{cGMP}]_{\rm dark}\varphi(0)\big\} dr_jdz \\
&\qquad + \sum_{j=1}^{m} \int_0^t \iint_{\mathcal{B}_j} r_j\theta_{j,\varepsilon_o}(r_j)\big\{ -[\mathrm{cGMP}]_{\mathcal{B}_j}\varphi_t + \mathrm{D}_{\rm cG}\nabla_{\mathcal{B}_j}[\mathrm{cGMP}]_{\mathcal{B}_j}\cdot\nabla_{\mathcal{B}_j}\varphi\big\} dr_jdzd\tau \Big\}_{\text{incisures}} = 0
\end{aligned}
$$

for all $t > 0$ and all smooth, real valued functions φ in $\bar{\Omega}\times\mathbb{R}^+$. Here

$$
\begin{aligned}
\alpha([\mathrm{Ca}^{2+}]) &= \alpha_{\min} + (\alpha_{\max}-\alpha_{\min})\frac{\mathrm{K}_{\rm cyc}^{\rm m_{cyc}}}{\mathrm{K}_{\rm cyc}^{\rm m_{cyc}} + [\mathrm{Ca}^{2+}]^{\rm m_{cyc}}} \\
\alpha([\mathrm{Ca}^{2+}]_*) &= \alpha_{\min} + (\alpha_{\max}-\alpha_{\min})\frac{\mathrm{K}_{\rm cyc}^{\rm m_{cyc}}}{\mathrm{K}_{\rm cyc}^{\rm m_{cyc}} + [\mathrm{Ca}^{2+}]_*^{\rm m_{cyc}}}
\end{aligned}
\qquad \text{where} \qquad
\begin{aligned}
\alpha_{\max} &= \mathrm{k}_{\rm GC,max}[\mathrm{GC}] \\
\alpha_{\min} &= \mathrm{k}_{\rm GC,min}[\mathrm{GC}].
\end{aligned}
$$

Here $\mathrm{k}_{\rm GC,min}$ and $\mathrm{k}_{\rm GC,max}$ are the minimum and maximum catalytic rates of production of cGMP by guanylyl cyclase GC occurring respectively as $[\mathrm{Ca}^{2+}] \to \infty$ and as $[\mathrm{Ca}^{2+}] \to 0$.

A.3 Weak Formulation of the Dynamics of Ca^{2+}

$$
\begin{aligned}
&(1-\mu_o)\Big\{ \iiint_{\Omega_{\rm eff}} \big\{[\mathrm{Ca}^{2+}](t)\varphi(t) - [\mathrm{Ca}^{2+}]_{\rm dark}\varphi(0)\big\} dxdydz \\
&\qquad + \int_0^t \iiint_{\Omega_{\rm eff}} \big\{ -[\mathrm{Ca}^{2+}]\varphi_t + \mathrm{D_{Ca}}\nabla_{(x,y)}[\mathrm{Ca}^{2+}]\cdot\nabla_{(x,y)}\varphi\big\} dxdydz\, d\tau \Big\}_{\rm interior} \\
&+\nu\varepsilon_o\Big\{ \sum_{i=1}^{k} \iint_{D^*_{i,\rm eff}} \big\{[\mathrm{Ca}^{2+}]_*(t)\varphi(t) - [\mathrm{Ca}^{2+}]_{\rm dark}\varphi(0)\big\} dxdy \\
&\qquad + \sum_{i=1}^{k}\int_0^t \iint_{D^*_{i,\rm eff}} \big\{ -[\mathrm{Ca}^{2+}]_*\varphi_t + \mathrm{D_{Ca}}\nabla_{(x,y)}[\mathrm{Ca}^{2+}]_*\cdot\nabla_{(x,y)}\varphi\big\} dxdy\, d\tau \Big\}_{\substack{\rm activated\\ \rm discs}} \\
&+\sigma\varepsilon_o\Big\{ \iint_S \big\{[\mathrm{Ca}^{2+}]_S(t)\varphi(t) - [\mathrm{Ca}^{2+}]_{\rm dark}\varphi(0)\big\} dS \\
&\qquad + \int_0^t \iint_S \big\{ -[\mathrm{Ca}^{2+}]_S\varphi_t + \mathrm{D_{Ca}}\nabla_S[\mathrm{Ca}^{2+}]_S\cdot\nabla_S\varphi\big\} dS\, d\tau \\
&\qquad + \int_0^t \iint_S \frac{1}{\sigma\varepsilon_o \mathrm{B_{Ca}}\mathcal{F}} \Big\{ \frac{\mathrm{j_{ex}^{sat}}}{\Sigma_{\rm rod}} \frac{[\mathrm{Ca}^{2+}]_S}{\mathrm{K_{ex}} + [\mathrm{Ca}^{2+}]_S} \\
&\qquad\qquad - \frac{1}{2}\mathrm{f_{Ca}} \frac{\mathrm{j_{cG}^{max}}}{\Sigma_{\rm rod}} \frac{[\mathrm{cGMP}]_S^{\mathrm{m_{cG}}}}{\mathrm{K_{cG}^{m_{cG}}} + [\mathrm{cGMP}]_S^{\mathrm{m_{cG}}}} \Big\} \varphi dS\, d\tau \Big\}_{\rm outer\ shell} \\
&+2\Big\{ \sum_{j=1}^{m} \iint_{\mathcal{B}_j} r_j\theta_{j,\varepsilon_o}(r_j) \big\{[\mathrm{Ca}^{2+}]_{\mathcal{B}_j}(t)\varphi(t) - [\mathrm{Ca}^{2+}]_{\rm dark}\varphi(0)\big\} dr_j dz \\
&+\sum_{j=1}^{m}\int_0^t \iint_{\mathcal{B}_j} r_j\theta_{j,\varepsilon_o}(r_j) \big\{ -[\mathrm{Ca}^{2+}]_{\mathcal{B}_j}\varphi_t + \mathrm{D_{Ca}}\nabla_{\mathcal{B}_j}[\mathrm{Ca}^{2+}]_{\mathcal{B}_j}\cdot\nabla_{\mathcal{B}_j}\varphi\big\} dr_j dz\, d\tau \Big\}_{\rm incisures} = 0
\end{aligned}
$$

for all $t > 0$ and all smooth, real valued functions φ in $\bar{\Omega} \times \mathbb{R}^+$.

A.4 Weak Formulation of the Dynamics of Transducer and Effector

$$
\begin{aligned}
\iint_{D_{\rm eff}} [\mathrm{T}^*](t)\varphi(t) dxdy + \int_0^t \iint_{D_{\rm eff}} \big\{ -[\mathrm{T}^*]\varphi_t + D_T\nabla[\mathrm{T}^*]\cdot\nabla\varphi \big\} dxdyd\tau \\
= \int_0^t k_\ell \varphi\big(x(\tau), y(\tau)\tau\big) d\tau - \int_0^t \iint_{D_{\rm eff}} \mathrm{k_{T^*E}}[\mathrm{E}][\mathrm{T}^*]\varphi dxdyd\tau
\end{aligned}
$$

$$
\begin{aligned}
\iint_{D_{\rm eff}} [\mathrm{E}^*](t)\varphi(t) dxdy + \int_0^t \iint_{D_{\rm eff}} \big\{ -[\mathrm{E}^*]\varphi_t + D_E\nabla[\mathrm{E}^*]\cdot\nabla\varphi \big\} dxdyd\tau \\
= \int_0^t \iint_{D_{\rm eff}} \big\{ \mathrm{k_{T^*E}}[\mathrm{E}][\mathrm{T}^*]\varphi - k_{\mathrm{E}^*}[\mathrm{E}^*]\varphi \big\} dxdyd\tau
\end{aligned}
$$

for all $t > 0$ and all smooth, real valued functions φ in $\bar{D}_R \times \mathbb{R}^+$.

B Appendix. Parameters

B.1 Mouse Parameters

Table S1: Parameters for the **Mouse** ROS

Symbol	Units	Definition	Value	References
$\alpha_{\max}$	μM/s	Maximum rate of cGMP synthesis at low Ca^{2+} concentration	76.5	[3, 66]
$\alpha_{\max}/\alpha_{\min}$	-	Suppression ratio of α from high to low Ca^{2+} concentration	13.9	[2, 3, 66]
A_{inc}	μm^2	Area of the incisure	0.0403	
β_{dark}	s^{-1}	Rate of cGMP hydrolysis by dark activated PDE	2.9	[8, 66]
B_{cG}	-	Buffering power of cytoplasm for cGMP	1	[53, 54]
B_{Ca}	-	Buffering power of cytoplasm for Ca^{2+}	20	[46, 47, 48]
c_{TE}	-	Coupling coefficient from T^* to E^*	1	[54, 34]
$[cGMP]_{dark}$	μM	Concentration of cGMP in the dark	3.80	[2, 30, 48, 51, 52, 53, 54, 66]
$[Ca^{2+}]_{dark}$	nM	Concentration of Ca^{2+} in the dark	344	[41, 71, 37, 16]
D_{cG}	$\mu m^2/s$	Diffusion coefficient of cGMP	120	[7, 24, 49]
D_{Ca}	$\mu m^2/s$	Diffusion coefficient of Ca^{2+}	15	[46]
D_{E^*}	$\mu m^2/s$	Diffusion coefficient of E^*	1.2	[53]
D_{T^*}	$\mu m^2/s$	Diffusion coefficient of T^*	2.2	[53]
D_{R^*}	$\mu m^2/s$	Diffusion coefficient of R^*	1.5	[53]
ε_o	nm	Disc thickness	14.5	[4, 19, 53]
η	nm	Volume-to-surface ratio	7.25	
$\mathcal{F}$	$\mathcal{C}mol^{-1}$	Faraday's constant	96500	
f_{Ca}	-	Fraction of $cGMP$-activated current carried by Ca^{2+}	0.06	[3, 39, 54, 57, 60]
H	μm	Height of ROS	23.6	[4, 36, 38, 15, 14, 31, 32]
j_{dark}	pA	Dark current	10.9	[2, 3, 8, 9, 18, 27, 28, 44, 54, 68, 70, 72]
j_{cG}^{max}	pA	Maximum CNG channel current	3550	
j_{ex}^{sat}	pA	Saturated exchanger current	1.8	[59, 61, 62]
k_{cat}/K_m	$\mu M^{-1}s^{-1}$	Hydrolytic efficiency of activated PDE dimer	540	[53, 34, 55]
$k_{\sigma;hyd}$	$\mu m^3/s$	Surface hydrolysis rate of cGMP by dark-activated PDE	2.8×10^{-5}	
$k^*_{\sigma;hyd}$	$\mu m^3/s$	Surface hydrolysis rate of cGMP by light-activated PDE	0.9	
k_E	s^{-1}	Rate constant for inactivation of PDE	6.5	[8, 25, 29, 39]
k_R	s^{-1}	Rate constant for inactivation of R^*	8.5	[8, 29, 47]
k_{T^*E}	$\mu m^2/s$	Kinetic constant of T^*-E formation and thus E^* production	1	[56]
K_{cyc}	nM	Half-saturating $[Ca^{2+}]$ for GC activity	100	[3, 66, 40, 42]
K_{cG}	μM	[cGMP] for half maximal CNG channel opening	20	[54]
K_{ex}	μM	$[Ca^{2+}]$ for half maximal exchanger rate	1.6	[54, 61]
ℓ_b	μm	Width of the incisure	0.2593	[11]
ℓ_r	μm	Length of the incisure	0.3111	[11]
ν	-	Ratio between interdiscal space and disc thickness	1	[4, 34, 53, 54]
$\nu\varepsilon_o$	nm	Interdiscal space	14.5	[4, 36, 38, 53, 20]

Continued on next page

Table S1 – continued from previous page

Symbol	Units	Definition	Value	References
ν_{RG}	s^{-1}	Rate of transducin formation per fully activated R*	330	[22]
n	-	Number of discs	814	
n_{inc}	-	Number of incisures	1	[4, 11, 53]
N_{Av}	$\#mol^{-1}$	Avogadro number	6.02×10^{23}	
m_{cyc}	-	Hill coefficient for GC effect	2	[2, 3, 8, 39, 66]
m_{cG}	-	Hill coefficient for CNG channels	3.5	[2, 8, 45, 53, 66]
PDE*	$\#\mu m^{-2}$	Surface density of dark-activated PDE	750	[19, 36, 53, 54, 65]
R	μm	Radius of disc	0.685	[4, 19, 33, 35, 36, 38, 53, 54]
σ	-	Ratio between outer shell thickness and disc thickness	15/14.5	
$\sigma\varepsilon$	nm	Distance between the disc rim and the plasma membrane (outer shell thickness)	15	[12, 13, 53, 20]
Σ_{rod}	μm^2	Lateral surface area of ROS	103.8	
V_{cyt}	μm^3	Cytoplasmic volume	18.16	

B.1.1 Mouse Deactivation Parameters in the Continuous Time Markov Chain (CTMC)

Determination of these parameters is in [5] and calibrated to ensure that the average lifetime $\tau_{\mathrm{R,eff}}$ of R* is $\frac{1}{2}t_{\mathrm{peak}}$. The value t_{peak} for $D_{\mathrm{cG}} = 330\mu m^2/s$ is essentially the same as for $D_{\mathrm{cG}} = 120\mu m^2/s$, so that the parameters in Table S2 based on $\tau_{\mathrm{R,eff}} \approx \frac{1}{2}t_{\mathrm{peak}}$ ([5]) remain unchanged for these two values of D_{cG}. In particular λ_o and μ_o were chosen to ensure that the average lifetime of R* is $\frac{1}{2}t_{\mathrm{peak}}$.

Table S2: **Mouse** CTMC Model Parameters

Symbol	Units	Definition	Value	References
λ_o	s^{-1}	Rhodopsin phosphorylation rate	10.5	[5]
μ_o	s^{-1}	Arrestin binding rate	60	[5]
k_ν	-	Decay constant of catalytic activity of R*	0.5	[69]
$\tau_{\mathrm{R,eff}}$	ms	Average lifetime of active R*	75	[29]
N	-	Average number of steps of R* before shut-off	4.45	[5]

B.2 Salamander Parameters

Table S3: Parameters for the **Salamander** ROS

Symbol	Units	Definition	Range	Value	References
$\alpha_{\max}$	μM/s	Maximum rate of cGMP synthesis at low Ca^{2+} concentration	40-50	50	[54, 48]
$\alpha_{\max}/\alpha_{\min}$	-	Ratio of $\alpha_{\max}$ to $\alpha_{\min}$	50	50	[54, 48]
A_{inc}	μm^2	Area of the incisure	0.82	0.8	[49]
β_{dark}	s^{-1}	Rate of $cGMP$ hydrolysis by dark activated PDE	1	1	[54, 48, 7, 6]
B_{cG}	-	Buffering power of cytoplasm for cGMP	1-2	1	[48, 53, 54]

Continued on next page

Table S3 – continued from previous page

Symbol	Units	Definition	Range	Value	References
B_{Ca}	-	Buffering power of cytoplasm for Ca^{2+}	10-50	20	[54, 48, 47]
c_{TE}	-	Coupling coefficient from T^* to E^*	< 1	1	[54, 34]
$[cGMP]_{dark}$	μM	Concentration of cGMP in the dark	2-4	3.0046	[48, 30]
$[Ca^{2+}]_{dark}$	nM	Concentration of Ca^{2+} in the dark	400-700	653.7	[48, 30]
D_{cG}	$\mu m^2/s$	Diffusion coefficient of $cGMP$	50-196	160	[7, 26, 49]
D_{Ca}	$\mu m^2/s$	Diffusion coefficient of Ca^{2+}	15	15	[46]
D_{E^*}	$\mu m^2/s$	Diffusion coefficient for activated PDE	0.8	0.8	[53]
D_{T^*}	$\mu m^2/s$	Diffusion coefficient for activated G protein	1.5	1.5	[53]
D_{R^*}	$\mu m^2/s$	Diffusion coefficient for R^*	0.7	0.7	[53]
ε_o	nm	Disc thickness	10-14	14	[54, 30]
η	nm	Volume-to-surface ratio		7	
$\mathcal{F}$	$\mathcal{C}mol^{-1}$	Faraday's constant	96500	96500	[54, 48]
f_{Ca}	-	Fraction of $cGMP$-activated current carried by Ca^{2+}	0.1-0.2	0.17	[54, 48]
H	μm	Height of ROS	19-26	22.4	[54, 23, 43, 10]
j_{dark}	pA	Dark current	74	65.862	[54]
j_{cG}^{max}	pA	Maximum cGMP-gated channel current	70-7000	7000	[48]
j_{ex}^{sat}	pA	Saturated exchanger current	17-20	17	[54]
k_{cat}/K_m	$\mu M^{-1}s^{-1}$	Hydrolytic efficiency of activated PDE dimer	340-600	400	[53, 48, 34]
$k_{\sigma;hyd}$	$\mu m^3/s$	Surface hydrolysis rate of $cGMP$ by dark-activated PDE		7×10^{-5}	[7]
$k^*_{\sigma;hyd}$	$\mu m^3/s$	Surface hydrolysis rate of $cGMP$ by light-activated PDE		0.5	[1, 7]
k_E	s^{-1}	Rate constant for inactivation of PDE	0.58-0.76	0.58	[47]
k_R	s^{-1}	Rate constant for inactivation of R^*	1.69-3.48	2.5	[47]
k_{T^*E}	$\mu m^2/s$	Kinetic constant describing the formation of $T^* - E$ complex and thus the production of E^*	1	1	[56]
K_{cyc}	nM	Half-saturating $[Ca^{2+}]$ for GC activity	100-230	135	[48, 30]
K_{cG}	μM	[cGMP] for half maximal CNG channel opening	13-32	20	[54, 48, 30]
K_{ex}	μM	$[Ca^{2+}]$ for half maximal exchanger rate	1.5-1.6	1.5	[48, 30]
ℓ_b	nm	Width of the incisure	10-12	15	[49]
ℓ_r	μm	Length of the incisure		4.6377	[6]
ν	-	Ratio between interdiscal space and disc thickness		1	
$\nu\varepsilon_o$	nm	Interdiscal space	10-14	14	[54, 30]
ν_{RG}	s^{-1}	Rate of transducin formation per fully activated R^*	120-220	185	[22, 34, 48, 54]
n	$-$	Number of discs	1000	800	[48, 30]
n_{inc}	$-$	Number of incisures	15-30	23	[17, 67, 50, 35, 58, 49]
N_{Av}	$\#mol^{-1}$	Avogadro number		6.02×10^{23}	
m_{cyc}	-	Hill coefficient for GC effect	2	2	[34, 54, 26]
m_{cG}	-	Hill coefficient for CNG channels	2-3	2.5	[54]
PDE^*	$\#\mu m^{-2}$	Surface density of dark-activated PDE	100	100	[54]
R	μm	Radius of disc	4.7-7.6	5.5	[23, 43, 64, 10]

Continued on next page

Table S3 – continued from previous page

Symbol	Units	Definition	Range	Value	References
σ	-	Ratio between outer shell thickness and disc thickness		15/14	[48, 30]
$\sigma\varepsilon$	nm	Distance between the disc rim and the plasma membrane (outer shell thickness)	15	15	[48, 30]
$\Sigma_{\rm rod}$	μm^2	Lateral surface area of ROS		773.5	[30]
$V_{\rm cyt}$	μm^3	Cytoplasmic volume	1000	1076	[54, 30]

B.2.1 Salamander Deactivation Parameters in the Continuous Time Markov Chain (CTMC)

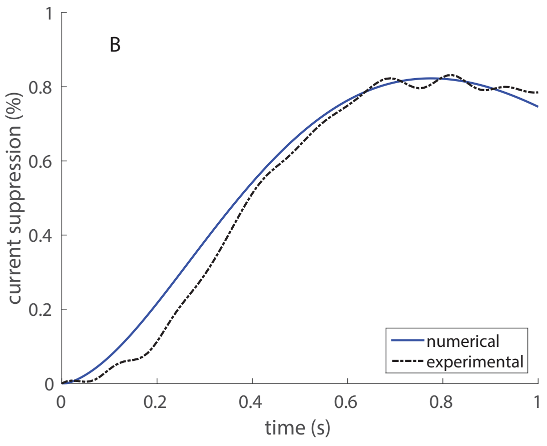

Figure S1: Simulation of the experimentally observed SPR of salamander kindly provided by F. Rieke as reported in [7] (black trace) using the FSR model with the parameters from Table S3 (blue trace).

These parameters are determined as in [5]. In particular λ_o and μ_o were chosen to ensure, as for mouse, that the average lifetime of R^* is $\frac{1}{2}t_{\rm peak}$. The parameters $k_\nu = 0.41$ and $\nu_{\rm RG} = 185/s$ were chosen to fit the experimental SPR curve reported in [7] and kindly provided by F. Rieke.

Table S4: **Salamander** CTMC Model Parameters

Symbol	Units	Definition	Value
λ_o	s^{-1}	Rhodopsin phosphorylation rate	2.0
μ_o	s^{-1}	Arrestin binding rate	10
k_ν	-	Rate of catalytic activity of R^*	0.41
$\tau_{\rm R,eff}$	s	Average lifetime of R^*	0.4
N	-	Average number of phosphorylation steps of R^* before full quench	4.45

C Appendix. Calibrating the Mouse Activation Parameter $\nu_{\rm RG}$ for $D_{\rm cG} = 330\mu m^2/s$

The model parameters for mouse, including the volumic diffusivity $D_{\rm cG} = 120\mu m^2/s$ were chosen and justified in [63, 5], and reported here in Table S1. The diffusivity $D_{\rm cG} = 330\mu m^2/s$ proposed in [21], was imported here

by keeping all the remaining parameters unchanged except the catalytic activity ν_{RG}, which was adjusted from $330\mathrm{s}^{-1}$ to $230\mathrm{s}^{-1}$ to reproduce the experimental SPR of [3, 5] (Figure S2).

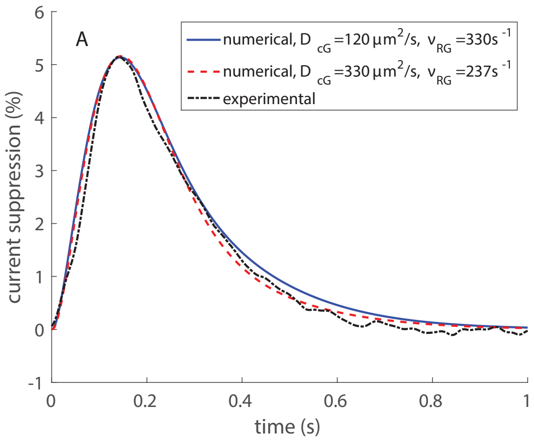

Figure S2: FSR simulations of the experimentally observed mouse rod SPR (black trace), from [3], using the parameters from Table S1 (red trace) and upon raising D_{cG} to $330\mu\mathrm{m}^2/\mathrm{s}$ with a concomitant lowering of ν_{RG} to $230\mathrm{s}^{-1}$ (blue trace).

D Appendix. Relating Volumic and Longitudinal Diffusivities

The longitudinal diffusivity $\mathrm{D}^{\ell}_{\mathrm{cG}}$ along the axis of the ROS, can be derived from the volumic D_{cG} by the formula $\mathrm{D}^{\ell}_{\mathrm{cG}} = (f_A/f_V)\mathrm{D}_{\mathrm{cG}}$, where f_A and f_V are two geometric parameters computed in [6] as

$$f_A = \frac{(\mathrm{A}_{\mathrm{inc}} + \mathrm{A}_{\mathrm{gap}})}{\pi R^2 + \mathrm{A}_{\mathrm{gap}}}; \qquad f_V = \frac{\pi R^2 + 2(\mathrm{A}_{\mathrm{inc}} + \mathrm{A}_{\mathrm{gap}})}{2(\pi R^2 + \mathrm{A}_{\mathrm{gap}})}$$

where $\mathrm{A}_{\mathrm{inc}}$ is the total cross-sectional area of the incisures, $\mathrm{A}_{\mathrm{gap}}$ is the cross-sectional area of the outer shell and R is the cross-sectional radius of the ROS.

References

[1] D. Andreucci, P. Bisegna, G. Caruso, H. E. Hamm, and E. DiBenedetto. Mathematical model of the spatio-temporal dynamics of second messengers in visual transduction. *Biophys. J.*, 85:1358–1376, 2003.

[2] M. E. Burns, A. Mendez, J. Chen, and D. A. Baylor. Dynamics of cyclic GMP synthesis in retinal rods. *Neuron*, 36:81–91, 2002.

[3] P. D. Calvert, V. I. Govardovskii, N. Krasnoperova, R. E. Anderson, J. Lem, and C. L. Makino. Membrane protein diffusion sets the speed of rod phototransduction. *Nature*, 411:90–94, 2001.

[4] L. D. Carter-Dawson and M. M. LaVail. Rods and cones in the mouse retina i. structural analysis using light and electron microscopy. *J. Comp. Neurol.*, 188:245–262, 1979.

[5] G. Caruso, P. Bisegna, L. Lenoci, D. Andreucci, V. V. Gurevich, H. E. Hamm, and E. DiBenedetto. Kinetics of rhodopsin deactivation and its role in regulating recovery and reproducibility of rod photoresponse. *PLoS Comput. Biol.*, 6(12):1–15, 2010. doi:10.1371/journal.pcbi.1001031.

[6] G. Caruso, P. Bisegna, L. Shen, D. Andreucci, H. E. Hamm, and E. DiBenedetto. Modeling the role of incisures in vertebrate phototransduction. *Biophys. J.*, 91:1192–1212, 2006.

[7] G. Caruso, H. Khanal, V. Alexiades, F. Rieke, H. E. Hamm, and E. DiBenedetto. Mathematical and computational modelling of spatio-temporal signalling in rod phototransduction. *I.E.E. Proc. Syst. Biol.*, 152:119–137, 2005.

[8] C. K. Chen, M. E. Burns, W. He, T. G. Wensel, D. A. Baylor, and M. I. Simon. Slowed recovery of rod photoresponse in mice lacking the GTPase accelerating protein RGS9-1. *Nature*, 403:557–560, 2000.

[9] C. K. Chen, M. E. Burns, M. Spencer, G. A. Niemi, J. Chen, J. B. Hurley, D. A. Baylor, and M. I. Simon. Abnormal photoresponses and light-induced apoptosis in rods lacking rhodopsin kinase. *Proc. Natl. Acad. Sci. U.S.A.*, 96:3718–3722, 1999.

[10] Y. Chen, S. Znoiko, W. J. DeGrip, R. K. Crouch, and J.-X. Ma. Salamander blue-sensitive cones lost during metamorphosis. *Photochem. Photobiol.*, 84:855–862, 2008.

[11] A. I. Cohen. The ultrastructure of the rods of the mouse retina. *Am. J. Anat.*, 107:23–48, 1960.

[12] J. M. Corless, R. D. Fetter, and M. J. Costello. Structural features of the terminal loop region of frog retinal rod outer segment disk membranes: I. Organization of lipid components. *J. Comp. Neurol.*, 257(1):1–8, 1987.

[13] J. M. Corless, R. D. Fetter, O. B. Zampighi, M. J. Costello, and D. L. Wall-Buford. Structural features of the terminal loop region of frog retinal rod outer segment disk membranes: II. Organization of the terminal loop complex. *J. Comp. Neurol.*, 257(1):9–23, 1987.

[14] G. H. Daly, J. M. Dileonardo, N. R. Balkema, and G. W. Balkema. The relationship between ambient lighting conditions, absolute dark-adapted thresholds, and rhodopsin in black and hyppopigmented mice. *Vis. Neurosci.*, 21:925–934, 2004.

[15] L. L. Daniele, C. Insinna, R. Chance, J. Wang, S. S. Nikonov, and E. N. Pugh Jr. A mouse M-opsin monochromat: retinal cone photoreceptors have increased M-opsin expression when S-opsin is knocked out. *Vision Res.*, 51:447–458, 2011.

[16] A. M. Dizhoor, M. L. Woodruff, E. V. Olshevskaya, M. C. Cilluffo, M. C. Cornwall, P. A. Sieving, and G. L. Fain. Night blindness and the mechanism of constitutive signaling of mutant G90D rhodopsin. *J. Neurosci.*, 28:11662–11672, 2008.

[17] M. S. Eckmiller. Microtubules in a rod-specific cytoskeleton associated with outer segment incisures. *Vis. Neurosci.*, 17:711–722, 2000.

[18] J. Fan, M. L. Woodruff, M. C. Cilluffo, R. K. Crouch, and G. L. Fain. Opsin activation of transduction in the rods of dark-reared Rpe65 knockout mice. *J. Physiol.*, 568:83–95, 2005.

[19] D. Fotiadis, Y. Liang, S. Filipek, D. A. Saperstein, A. Engel, and K. Palczewski. Atomic force microscopy: rhodopsin dimers in native disc membranes. *Nature*, 421:127–128, 2003.

[20] J. C. Gilliam, J. T. Chang, I. M. Sandoval, Y. Zhang, T. Li, S. J. Pittler, W. Chiu, and T. G. Wensel. Three-dimensional architecture of the rod sensory cilium and its disruption in retinal neurodegeneration. *Cell*, 151(5):1029–1041, Nov 2012.

[21] O. P. Gross, E. N. Pugh Jr., and M. E. Burns. Spatiotemporal cGMP dynamics in living mouse rods. *Biophys. J.*, 102(8):1775–1784, 2012.

[22] R. D. Hamer, S. C. Nicholas, D. Tranchina, P. A. Liebman, and T. D. Lamb. Multiple steps of phosphorylation of activated rhodopsin can account for the reproducibility of vertebrate rod single-photon responses. *J. Gen. Physiol.*, 122:419–444, 2003.

[23] F. I. Harosi. Absorption spectra and linear dichroism of some amphibian photoreceptors. *J. Gen. Physiol.*, 66:357–382, 1975.

[24] D. Holcman and J. I. Korenbrot. Longitudinal diffusion in retinal rod and cone outer segment cytoplasm: the consequence of cell structure. *Biophys. J.*, 86:2566–2582, 2004.

[25] M. J. Kennedy, M. E. Sowa, T. G. Wensel, and J. B. Hurley. Acceleration of key reactions as a strategy to elucidate the rate-limiting chemistry underlying phototransduction inactivation. *Invest. Ophthalmol. Vis. Sci.*, 44:1016–1022, 2003.

[26] Y. Koutalos, K. Nakatani, and K. W. Yau. Cyclic GMP diffusion coefficient in rod photoreceptor outer segments. *Biophys. J.*, 68:373–382, 1995.

[27] C. M. Krispel, C. K. Chen, M. I. Simon, and M. E. Burns. Novel form of adaptation in mouse retinal rods speeds recovery of phototransduction. *J. Gen. Physiol.*, 122:703–712, 2003.

[28] C. M. Krispel, C. K. Chen, M. I. Simon, and M. E. Burns. Prolonged photoresponses and defective adaptation in rods of Gbeta5-/- mice. *J. Neurosci.*, 23:6965–6971, 2003.

[29] C. M. Krispel, D. Chen, N. Melling, Y. J. Chen, K. A. Martemyanov, N. Quillinan, V. Y. Arshavsky, T. G. Wensel, C. K. Chen, and M. E. Burns. RGS expression rate-limits recovery of rod photoresponses. *Neuron*, 51:409–416, 2006.

[30] T. D. Lamb and E. N. Pugh Jr. A quantitative account of the activation steps involved in phototransduction in amphibian photoreceptors. *J. Physiol.*, 449:719–758, 1992.

[31] M. M. LaVail. Kinetics of rod outer segment renewal in the developing mouse retina. *J. Cell. Biol.*, 58:650–661, 1973.

[32] M. M. LaVail, M. P. White, G. M. Gorrin, D. Yasumura, K. V. Porrello, and R. J. Mullen. Retinal degeneration in the nervous mutant mouse. I. Light microscopic cytopathology and changes in the interphotoreceptor matrix. *J. Comp. Neurol.*, 333:168–181, 1993.

[33] J. Lem, N. V. Krasnoperova, P. D. Calvert, B. Kosaras, D. A. Cameron, M. Nicolo, C. L. Makino, and R. L. Sidman. Morphological, physiological, and biochemical changes in rhodopsin knockout mice. *Proc. Natl. Acad. Sci. U. S. A.*, 96:736–741, 1999.

[34] I. B. Leskov, V. A. Klenchin, J. W. Handy, G. G. Whitlock, V. I. Govardovskii, M. D. Bownds, T. D. Lamb, E. N. Pugh Jr., and V. Y. Arshavsky. The gain of rod phototransduction: reconciliation of biochemical and electrophysiological measurements. *Neuron*, 27:525–537, 2000.

[35] Y. Liang, D. Fotiadis, S. Filipek, D. A. Saperstein, K. Palczewski, and A. Engel. Organization of the G protein-coupled receptors rhodopsin and opsin in native membranes. *J. Biol. Chem.*, 278:21655–21662, 2003.

[36] Y. Liang, D. Fotiadis, T. Maeda, A. Maeda, A. Modzelewska, S. Filipek, D. A. Saperstein, A. Engel, and K. Palczewski. Rhodopsin signaling and organization in heterozygote rhodopsin knockout mice. *J. Biol. Chem.*, 279:48189–48196, 2004.

[37] X. Liu, O. V. Bulgakov, X. H. Wen, M. L. Woodruff, B. Pawlyk, J. Yang, G. L. Fain, M. A. Sandberg, C. L. Makino, and T. Li. AIPL1, the protein that is defective in Leber congenital amaurosis, is essential for the biosynthesis of retinal rod cGMP phosphodiesterase. *Proc. Natl. Acad. Sci. U.S.A.*, 101(38):13903–13908, 2004.

[38] A. L. Lyubarsky, L. L. Daniele, and E. N. Pugh Jr. From candelas to photoisomerizations in the mouse eye by rhodopsin bleaching in situ and the light-rearing dependence of the major components of the mouse ERG. *Vision Res.*, 44:3235–3251, 2004.

[39] C. L. Makino, R. L. Dodd, J. Chen, M. E. Burns, A. Roca, M. I. Simon, and D. A. Baylor. Recoverin regulates light-dependent phosphodiesterase activity in retinal rods. *J. Gen. Physiol.*, 123:729–741, 2004.

[40] C. L. Makino, I. V. Peshenko, X.-H. Wen, E. V. Olshevskaya, and R. Barrett. A role for GCAP2 in regulating the photoresponse. guanylyl cyclase activation ad rod electrophysiology in GUCA 1B knock-out mice. *J. Biol. Chem.*, 283(43):29135–29143, 2008.

[41] C. L. Makino, X.-H. Wen, N. Michaud, I. V. Peshenko, B. Pawlyk, R. S. Brush, M. Soloviev, X. Liu, M. L. Woodruff, P. D. Calvert, A. B. Savchenko, R. E. Anderson, G. L. Fain, T. Li, M. A. Sandberg, and A. M. Dizhoor. Effects of low AIPL1 expression on phototransduction in rods. *Invest. Ophthalmol. Vis. Sci.*, 47(5):2185–2194, 2006.

[42] C. L. Makino, X. H. Wen, E. V. Olshevskaya, I. V. Peshenko, A. B. Savchenko, and A. M. Dizhoor. Enzymatic relay mechanism stimulates cyclic GMP synthesis in rod photoresponse: biochemical and physiological study in guanylyl cyclase activating protein 1 knockout mice. *PLoS ONE*, 7(10):e47637, 2012.

[43] A. P. Mariani. Photoreceptors of the larval tiger salamander. *Proc. Roy. Soc. Lond.*, B 227:483–492, 1986.

[44] A. Mendez, M. E. Burns, A. Roca, J. Lem, L. W. Wu, M. I. Simon, D. A. Baylor, and J. Chen. Rapid and reproducible deactivation of rhodopsin requires multiple phosphorylation sites. *Neuron*, 28:153–164, 2000.

[45] A. Mendez and J. Chen. Mouse models to study GCAP functions in intact photoreceptors. *Adv. Exp. Med. Biol.*, 514:361–388, 2002.

[46] K. Nakatani, C. Chen, and Y. Koutalos. Calcium diffusion coefficient in rod photoreceptor outer segments. *Biophys. J.*, 82(2):728–739, 2002.

[47] S. Nikonov, N. Engheta, and E. N. Pugh Jr. Kinetics of recovery of the dark-adapted salamander rod photoresponse. *J. Gen. Physiol.*, 111:7–37, 1998.

[48] S. Nikonov, T. D. Lamb, and E. N. Pugh Jr. The role of steady phosphodiesterase activity in the kinetics and sensitivity of the light-adapted salamander rod photoresponse. *J. Gen. Physiol.*, 116:795–824, 2000.

[49] A. Olson and E. N. Pugh Jr. Diffusion coefficient of cyclic GMP in salamander rod outer segments estimated with two fluorescent probes. *Biophys. J.*, 65:1335–1352, 1993.

[50] D. S. Papermaster, P. Reilly, and B. G. Schneider. Cone lamellae and red and green rod outer segment disks contain a large intrinsic membrane protein on their margins: an ultrastructural immunocytochemical study of frog retinas. *Vision Res.*, 22:1417–1428, 1982.

[51] E. N. Pugh Jr., T. Duda, A. Sitaramayya, and R. K. Sharma. Photoreceptor guanylate cyclases: a review. *Biosci. Rep.*, 17:429–473, 1997.

[52] E. N. Pugh Jr. and T. D. Lamb. Cyclic GMP and calcium: the internal messengers of excitation and adaptation in vertebrate photoreceptors. *Vision Res.*, 30:1923–1948, 1990.

[53] E. N. Pugh Jr. and T. D. Lamb. Amplification and kinetics of the activation steps in phototransduction. *Biochim. Biophys. Acta*, 1141:111–149, 1993.

[54] E. N. Pugh Jr. and T. D. Lamb. *Phototransduction in vertebrate rods and cones: molecular mechanisms of amplification, recovery and light adaptation*, volume 3 of *Handbook of Biological Physics*, chapter 5, pages 183–255. Elsevier Science, 2000.

[55] N. Qin and W. Baehr. Expression and mutagenesis of mouse rod photoreceptor cGMP phosphodiesterase. *J. Biol. Chem.*, 269:3265–3271, 1994.

[56] S. Ramanathan, P. B. Detwiler, A. M. Sengupta, and B. I. Shraiman. G-protein-coupled enzyme cascades have intrinsic properties that improve signal localization and fidelity. *Biophys. J.*, 88:3063–3071, 2005.

[57] H. Reilander, A. Achilles, U. Friedel, G. Maul, F. Lottspeich, and N. J. Cook. Primary structure and functional expression of the Na/Ca,K-exchanger from bovine rod photoreceptors. *EMBO J.*, 11:1689–1695, 1992.

[58] D. Roof, M. Adamian, D. Jacobs, and A. Hayes. Cytoskeletal specializations at the rod photoreceptor distal tip. *J. Comp. Neurol.*, 305:289–303, 1991.

[59] P. P. Schnetkamp. Na-Ca or Na-Ca-K exchange in rod photoreceptors. *Prog. Biophys. Mol. Biol.*, 54:1–29, 1989.

[60] P. P. Schnetkamp. Cation selectivity of and cation binding to the cGMP-dependent channel in bovine rod outer segment membranes. *J. Gen. Physiol.*, 96:517–534, 1990.

[61] P. P. Schnetkamp. Optical measurements of Na-Ca-K exchange currents in intact outer segments isolated from bovine retinal rods. *J. Gen. Physiol.*, 98:555–573, 1991.

[62] P. P. Schnetkamp, R. T. Szerencsei, and D. K. Basu. Unidirectional Na^+, Ca^{2+}, and K^+ fluxes through the bovine rod outer segment Na-Ca-K exchanger. *J. Biol. Chem.*, 266:198–206, 1991.

[63] L. Shen, G. Caruso, P. Bisegna, D. Andreucci, V. V. Gurevich, H. E. Hamm, and E. DiBenedetto. Dynamics of mouse rod phototransduction and its sensitivity to variation of key parameters. *IET Syst. Biol.*, 4:12–32, 2010.

[64] D. M. Sherry, D. D. Bui, and W. J. DeGrip. Identification and distribution of photoreceptor subtypes in neotenic salamander retina. *Vis. Neurosci.*, 15:1175–1187, 1998.

[65] A. Sitaramayya, J. Harkness, J. H. Parkes, C. Gonzalez-Oliva, and P. A. Liebman. Kinetic studies suggest that light-activated cyclic GMP phosphodiesterase is a complex with G-protein subunits. *Biochemistry*, 25:651–656, 1986.

[66] S. H. Tsang, M. E. Burns, P. D. Calvert, P. Gouras, D. A. Baylor, S. P. Goff, and V. Y. Arshavsky. Role for the target enzyme in deactivation of photoreceptor G protein in vivo. *Science*, 282:117–121, 1998.

[67] Y. Tsukamoto. The number, depth and elongation of disc incisures in the retinal rod of Rana catesbeiana. *Exp. Eye Res.*, 45:105–116, 1987.

[68] Z. Wang, X. H. Wen, Z. Ablonczy, R. K. Crouch, C. L. Makino, and J. Lem. Enhanced shutoff of phototransduction in transgenic mice expressing palmitoylation-deficient rhodopsin. *J. Biol. Chem.*, 280:24293–24300, 2005.

[69] U. Wilden. Duration and amplitude of the light-induced cGMP hydrolysis in vertebrate photoreceptors are regulated by multiple phosphorylation of rhodopsin and by arrestin binding. *Biochemistry*, 34:1446–1454, 1995.

[70] M. L. Woodruff, J. Lem, and G. L. Fain. Early receptor current of wild-type and transducin knockout mice: photosensitivity and light-induced Ca^{2+} release. *J. Physiol.*, 557:821–828, 2004.

[71] M. L. Woodruff, A. P. Sampath, H. R. Matthews, N. V. Krasnoperova, J. Lem, and G. L. Fain. Measurement of cytoplasmic calcium concentration in the rods of wild-type and transducin knock-out mice. *J. Physiol.*, 542:843–854, 2002.

[72] J. Xu, R. L. Dodd, C. L. Makino, M. I. Simon, D. A. Baylor, and J. Chen. Prolonged photoresponses in transgenic mouse rods lacking arrestin. *Nature*, 389:505–509, 1997.
